# Comparison of the predictive performance of systemic immune-inflammation index and neutrophil-to-lymphocyte ratio for three-month poor functional outcome in ischemic stroke: a systematic review and meta-analysis

**DOI:** 10.1080/07853890.2026.2612820

**Published:** 2026-01-12

**Authors:** Xuan Ma, Yinjuan Zhou, Zhenhua Li, Guoming Mao, Haiping Wei, Tingting Zhao

**Affiliations:** ^a^ The Second Clinical Medical School, Lanzhou University, Lanzhou, Gansu, PR China; ^b^ Department of Neurology, Lanzhou University Second Hospital, Lanzhou, Gansu, PR China; ^c^ Department of Psychology, Lanzhou University Second Hospital, Lanzhou, Gansu, PR China

**Keywords:** Ischaemic stroke, neutrophils, lymphocytes, platelets, prognosis

## Abstract

**Introduction:**

Ischemic stroke (IS) is a leading cause of global mortality and disability. Early and accurate prognosis is crucial for patient management. The neutrophil-to-lymphocyte ratio (NLR) and systemic immune-inflammation index (SII) are emerging inflammatory biomarkers; however, their relative predictive value for three-month poor functional outcome (modified Rankin Scale [mRS] > 2) remains uncertain.

**Methods:**

We systematically searched PubMed, Embase, Web of Science, and the Cochrane Library up to 20 July 2025, adhering to PRISMA guidelines. Observational studies reporting the association of SII or NLR with three-month poor outcome were included. Study quality was evaluated using the Newcastle-Ottawa Scale. Area under the curve (AUC), odds ratios (OR), and standardized mean differences (SMD) were pooled using random-effects models in Stata 16.0.

**Results:**

Twenty-one studies involving 7520 IS patients were analysed. NLR demonstrated marginally superior discriminative ability compared to SII (AUC 0.71, 95% CI: 0.67–0.76 vs. 0.68, 95% CI: 0.64–0.71), though this difference was not statistically significant. Elevated NLR was significantly associated with poor outcome (OR = 1.26, 95% CI: 1.17–1.37, *p* < .001), whereas SII was not (OR = 1.00, 95% CI: 1.00–1.00, *p* = .384). Both markers showed moderate effect sizes (SMD: NLR = 0.69, SII = 0.72; *p* < .001). NLR performed better in non-intervention and Chinese subgroups, while SII exhibited consistent AUC values across treatment and ethnic subgroups.

**Conclusion:**

NLR and SII are accessible prognostic markers in IS. NLR demonstrates superior accuracy and a significant association with poor outcome, while SII shows greater stability across patient subgroups. Both may assist in risk stratification, in resource-limited settings.

## 1. Introduction

Stroke is the second leading cause of death globally and the third leading cause of death and disability among non-communicable diseases, with its burden rising most sharply in low- and lower-middle-income countries (LMICs) from 1990 to 2021 [1]. Ischaemic Stroke (IS), the predominant subtype of cerebrovascular disease, accounts for approximately 62.4% of all global stroke cases [2], and its disease burden continues to rise.2017 data showed that globally, there were 11.9 million new stroke cases, with IS comprising approximately 87% (about 10.35 million cases) [3]. By 2030, new IS cases are projected to exceed 15 million [4], straining public health systems. Despite declining age-standardized mortality rates, absolute deaths from IS reached 3.29 million in 2019 and are expected to rise to 4.90 million by 2030 [5]. Timely identification of high-risk groups and optimized prognosis assessment are thus critical to reducing disability, mortality, and associated healthcare costs.

Mounting evidence indicates that inflammation plays a dual role in ischaemic brain injury – harm then repair. Li et al. described an early phase (hours to days) featuring neutrophil infiltration and pro-inflammatory cytokine release, exacerbating blood–brain barrier disruption and neuronal apoptosis. Later (days to weeks), the response transitions to lymphocyte-mediated repair. Imbalance in this process directly affects infarct volume, neurological deficit severity, and long-term prognosis [6]. The neutrophil-to-lymphocyte ratio (NLR), a well-established inflammatory marker, is consistently associated with neurological deficit severity after stroke. A 2021 meta-analysis by Li et al. confirmed that elevated NLR correlates with adverse outcomes in ischaemic stroke, including increased mortality and higher likelihood of modified Rankin Scale (mRS) scores ≥3, indicating poorer prognosis. These findings support NLR as a useful prognostic biomarker in ischaemic stroke [7]. The Systemic Immune-Inflammation Index (SII), derived from platelet, neutrophil, and lymphocyte counts (P × N/L), better reflects the interplay between systemic inflammation and immune suppression. A 2023 study by Huang et al. of 234 acute ischaemic stroke patients found that elevated SII was independently associated with poor functional outcome (mRS score ≥2) at three months (OR = 2.35, 95% CI: 1.149–4.803, *p* = .019). Its predictive performance was comparable to NLR (AUC = 0.678 vs. 0.682) [8].

However, we specifically selected NLR and SII for comparison given their shared advantage of being derived from routine blood tests – cost-effective and accessible for resource-constrained settings – and their ability to capture complementary pathophysiological dimensions: NLR reflects neuroinflammation *via* neutrophil-lymphocyte balance, while SII integrates platelets to reflect inflammation-thrombosis crosstalk. Although both indices have been demonstrated to be associated with stroke severity and prognosis, direct comparative evidence regarding their predictive performance remains limited, and existing correlative data have not been systematically consolidated. This meta-analysis aims to systematically compare the predictive performance of SII and NLR for poor functional outcome (defined as [mRS] score > 2) at three months in patients with ischaemic stroke. By conducting a head-to-head comparison of their core performance indicators, this study seeks to provide evidence-based guidance for clinicians in selecting the most appropriate inflammatory biomarker. Additionally, the findings may offer valuable insights for prognostic stratification management in resource-constrained settings.

## 2. Methods

### 2.1. Aims and PICO statement

This study is a systematic review and meta-analysis of previously published literature. Therefore, ethical approval from an institutional review board and patient consent were not required. Our study strictly complied with the PRISMA (the Preferred Reporting Items for Systematic Reviews and Meta-Analyses) statement (Supplementary Table 1). We registered our study at PROSPERO with the identifier CRD420251128123 (https://www.crd.york.ac.uk/PROSPERO/view/CRD420251128123) on 17 August 2025.These were the PICO statements: (1) Population (P): Patients diagnosed with ischaemic stroke according to international diagnostic criteria (e.g. WHO criteria or TOAST classification), irrespective of age, sex, time since stroke onset, or aetiological subtype. Patients receiving any form of treatment were included. (2) Intervention/Index Predictor (I): SII as the predictor of interest, calculated as Platelet count × Neutrophil count/Lymphocyte count. (3) Comparison (C): NLR as the comparator predictor, calculated as Neutrophil count/Lymphocyte count. (4) Outcome (O): The primary outcome was poor functional outcome, defined as mRS score > 2 at three months post-stroke onset. Effect measures included:① Diagnostic performance metrics: Area Under the Curve (AUC) with its 95% Confidence Interval (95% CI). ② Association strength measures: Odds Ratio (OR) with its 95% CI. ③ Between-group difference measures: Standardized Mean Difference (SMD) with its 95% CI.

### 2.2. Literature search strategy

A comprehensive literature search was performed using both electronic database searching and manual retrieval. The search included four major biomedical databases: PubMed, Embase, Web of Science, and the Cochrane Library (CENTRAL). Search terms were categorized into three groups: (1) disease-related terms: ‘ischaemic stroke’, ‘cerebral infarction’, ‘acute ischemic stroke’; (2) index-related terms: ‘systemic immune-inflammation index’, ‘SII’, ‘neutrophil-to-lymphocyte ratio’, ‘NLR’, ‘platelet* neutrophil* lymphocyte’; and (3) outcome-related terms: ‘prognosis’, ‘outcome’, ‘modified Rankin Scale’, ‘mRS’, ‘functional outcome’, ‘3-month outcome’. The strategy combined Medical Subject Headings (MeSH)/Emtree terms and free-text keywords to maximize sensitivity and specificity. Searches covered records from database inception until 20 July 2025. To minimize omission bias, supplementary searches were conducted through backward citation tracking of included studies and relevant reviews. Two investigators independently executed the search process, cross-checked results, and resolved discrepancies through consensus or third-party adjudication when necessary.

### 2.3. Inclusion and exclusion criteria

Studies were included if they comprised patients with confirmed ischaemic stroke per international criteria (e.g. AHA/ASA or CT/MRI confirmation); reported association between SII or NLR and three-month mRS score with extractable/calculable effect measures (AUC, OR, or SMD, each with 95% CI); explicitly defined poor outcome as mRS > 2 (i.e. mRS 3–6) with clear assessment methodology, strictly adhering to the predefined threshold – two independent investigators verified the consistency of this outcome definition for each candidate study, with cross-checking to exclude studies using alternative thresholds or ambiguous outcome criteria; were peer-reviewed observational studies.

Studies were excluded if they involved animal, *in vitro*, or non-original research; lacked extractable three-month mRS outcome data or quantifiable effect measures (AUC, OR, SMD, with 95% CIs); defined poor outcome using alternative mRS thresholds (e.g. mRS ≥ 2, mRS > 3, mRS ≥ 3) instead of the predefined mRS> 2; were duplicate publications; were published in languages other than English; or omitted adjustment for critical confounders without feasibility for subgroup correction.

### 2.4. Data extraction

Two trained investigators independently conducted literature screening and data extraction using a standardized form, recording general study characteristics (e.g. author, year, country, design, sample size), patient baseline features (age, sex, stroke subtype), predictor-related data (timing and method of SII/NLR measurement), and outcome metrics (mRS definition, assessment protocol, follow-up, AUC/OR with 95% CI, median and IQR by outcome group).

For studies with missing data or ambiguous reporting, corresponding authors were contacted *via* email to request supplementary information. Studies were classified as having incomplete data and excluded if no response was received within two weeks. The two primary investigators (X. Ma and Y. Zhou) independently extracted all data. A third investigator (Z. Li) subsequently verified all extracted entries. Any discrepancies were resolved through consensus meetings involving all three researchers.

### 2.5. Risk of bias evaluation

Methodological quality of the included observational studies was assessed using the Newcastle-Ottawa Scale (NOS), which evaluates studies across three domains – selection (up to 4 points), comparability (up to 2 points), and outcome assessment (up to 3 points) – yielding a total score ranging from 0 to 9; studies scoring ≥6 points were considered high quality. Two investigators independently performed all assessments, with any discrepancies resolved through consensus discussion.

### 2.6. Statistical analysis

All statistical analyses were performed using Stata 16.0 (StataCorp, College Station, TX). The bivariate random-effects model was used to pool AUC values with 95% CIs for SII and NLR, incorporating sensitivity-specificity correlation for improved diagnostic performance estimation, with between-AUC differences assessed *via* Z-tests (*p* < .05 deemed significant). For dichotomous poor functional outcomes, ORs with 95% CIs were pooled using the DerSimonian-Laird random-effects model, with fixed-effects Mantel-Haenszel method applied when I^2^ < 50%. For between-group differences, means and standard deviations were estimated from medians and interquartile ranges using established methods (Luo et al. [9] and Wan et al. [10]) to compute SMDs with 95% CIs comparing good (mRS ≤2) and poor (mRS >2) outcome groups. SMDs with 95% CIs were pooled using a random-effects model. Heterogeneity was evaluated with the Higgins I^2^ statistic (0%–25% = negligible; 25%–50% = low; 50%–75% = moderate; >75% = high), with significance defined as *p* < .10 on Cochran’s Q-test or I^2^ > 50% [11]. When substantial heterogeneity existed, subgroup analyses were conducted to explore potential sources.

Publication bias was assessed using Begg’s and Egger’s tests, with *p* < .10 considered significant. Leave-one-out sensitivity analysis was conducted by sequentially excluding each study and recalculating pooled effect sizes with 95% CIs using the original statistical models. Instability was defined as *a* > 10% change in effect magnitude.

## 3. Results

### 3.1. Characteristics of included studies

**Figure 1.**
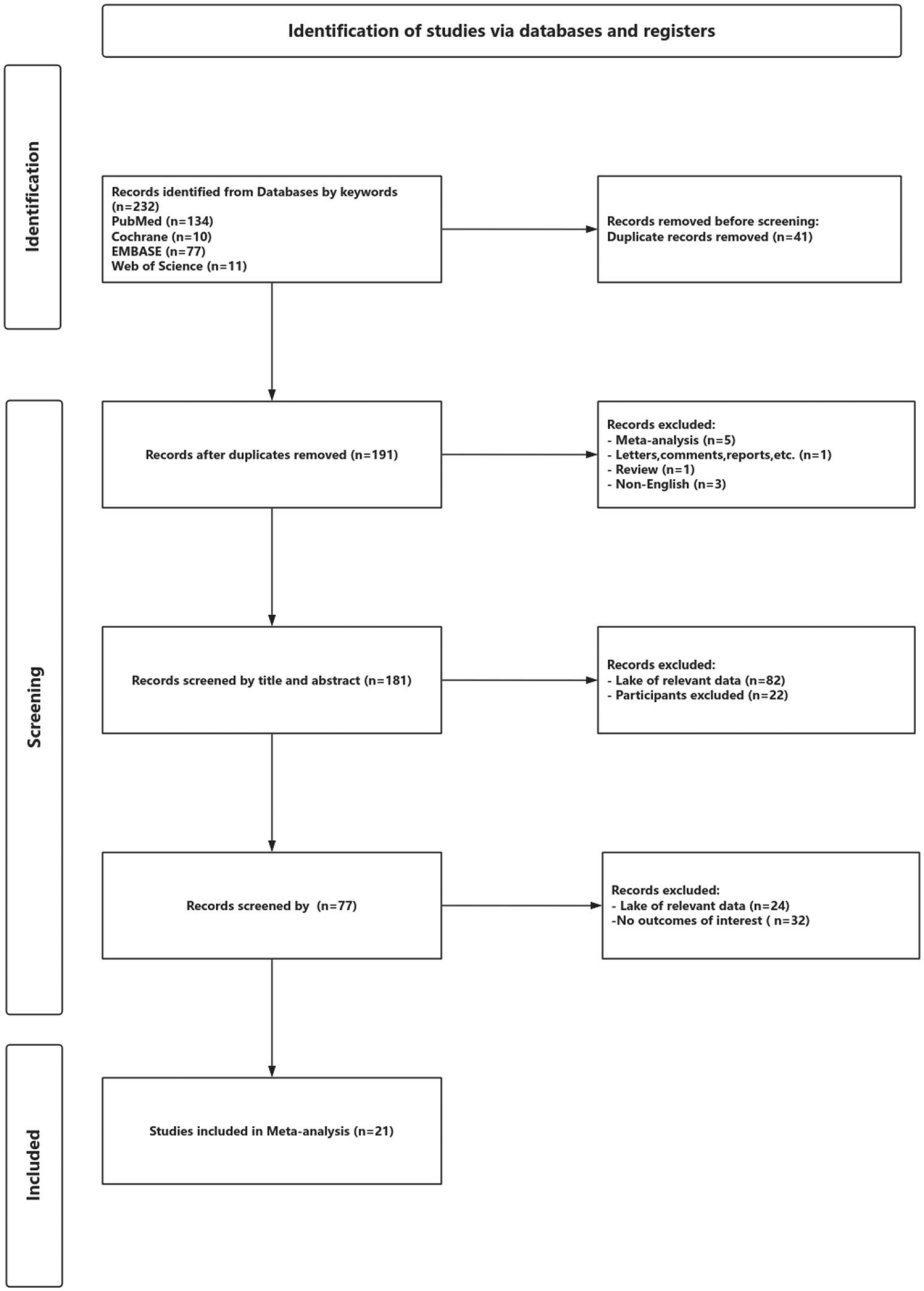
The study screening flowchart.

A total of 232 articles were identified through database searches and manual retrieval. After removing 41 duplicates, 114 records were excluded through title and abstract screening. 77 articles underwent full-text assessment, of which 56 were excluded due to incomplete data, mismatched outcome definitions, or duplication, yielding 21 articles included for analysis (see Figure 1 for the study selection flowchart). These studies comprised 18 retrospective cohort studies, two prospective observational cohort studies, and one retrospective case-control study [8,12–31], originating from three countries: China (*n* = 17), United Kingdom, Italy, United States (*n* = 1), Turkey (*n* = 2), and South Korea (*n* = 1). The total sample size was 7520 patients (SII cohort: 2948; NLR cohort: 6029). All studies demonstrated moderate to high quality on the Newcastle–Ottawa Scale (scores 6–9). Key characteristics, quality assessments, and patient demographics are detailed in Table 1.

**Table 1.** Baseline characteristics of the included studies and patients.

Author	Year	Nation	Study design	Participants (n)	Age(y)	Time of blood sample	Laboratory test method	Type of intervention	Primary endpoints	Follow-up time	SII AUC(95%CI)	SII median (IQR)	SII OR (95%CI)	NLR AUC (95%CI)	NLR median (IQR)	NLR OR (95%CI)	NOS
Ying et al.	2021	China	SC-RCS	899	61 ± 7^a^	On admission	Routine blood	EVT+IVT	mRS > 2 at 3 months	3 months	–	–	–	0.613(0.575–0.650)***	Poor outcome: 3.18 (2.25–4.53), good outcome: 2.60 (1.88–3.54)***	–	8
Wu et al.	2025	China	RCS	97	45.3 ± 9.1^a^	On admission	Routine blood	No surgery intervention	mRS > 2 at 3 months	3 months (±14 days)	0.667 (0.537–0.797)*	Poor outcome: 708.5 (466.8–1215.9), good outcome: 512.0 (348.8–686.3)*	–	–	Poor outcome: 2.2 (1.8–4.0), good outcome: 2.1(1.6–2.8) *p* = 0.123	–	6
Zhu et al.	2024	China	RCS	306	75.327 ± 8.911^a^	On admission	Routine blood	No surgery intervention	mRS > 2 at 3 months	3 months	–	Poor outcome: 770.868 (597.277–1092.731), good outcome: 447.552 (363.871–643.848)***	0.996(0.994–0.999)***	0.745(0.686–0.804)***	Poor outcome: 3.637 (2.886–5.289), good outcome: 2.290 (1.652–3.349)***	1.334(1.038–1.715)***	8
Xu et al.	2023	China	PCS	341	SP:68.81 ± 13.00 Non-SP:67.73 ± 11.82^a^	On admission	Routine blood	EVT+IVT	mRS > 2 at 3 months	3 months (±14 days)	–	–	–	0.6117(0.5341–0.6893)**	–	1.071(1.016–1.128)**)	9
Topcuoglu et al.	2021	Turkey	RCS	165	70 ± 14^a^	Pre/post-treatment 24h	Full blood	No surgery intervention	mRS > 2 at 3 months	3 months	0.688(0.611–0.758)***	–	–	0.712(0.636–0.780)***	–	–	7
Zhou et al.	2025	China	RCS	278	74 (23–92)	On admission	Full blood	EVT+IVT	mRS > 2 at 3 months, MCE	3 months	0.698(0.637–0.760)***	Poor outcome: 1269.83 (750.82–2497.22), good outcome: 422.33 (258.69–624.68)***	1.001(1.001–1.002)***	0.694(0.632–0.756)***	Poor outcome: 4.76 (2.59–7.72), good outcome: 2.73 (1.68–4.40)***	18.207(5.738–57.772)***	9
Qun et al.	2017	China	RCS	143	70 (54–86)	On admission	Full blood	No surgery intervention	mRS > 2 at 3 months	3 months	–	–	–	0.84(0.77–0.81)***	–	2.547(1.567–4.137)***	8
Wang et al.	2023	China	RCS	717	68 (58–75)	On admission(pre-thrombolysis)	Full blood	EVT	mRS > 2 at 3 months	3 months	0.598(0.552–0.645)***	Poor outcome: 738.76 (488.59–1270.92), good outcome: 558.01(345.60–907.26)***	–	0.617(0.570–0.663)***	Poor outcome: 3.87 (2.39–6.56), good outcome: 2.87 (1.81–4.76)***	–	9
Zhu et al.	2023	China	RCS	136	73.907 ± 9.990^a^	Within 24h of admission	Full blood	No surgery intervention	mRS > 2 at 3 months	3 months	–	–	1.061(1.031–1.092)***	0.719(0.6270–0.8108)***	Poor outcome: 3.593 (2.755–5.197), good outcome: 2.259 (1.715–3.372)***	1.252(1.008–1.554)*	9
Sun et al.	2025	China	RCS	841	70 ± 8.5^a^	On admission	Full blood	EVT+IVT	mRS > 2 at 3 months	3 months	0.725(0.676–0.774)***	Poor outcome: 1036.33 (680–1575.41), good outcome: 592.57 (399.12–925.68)***	–	0.730(0.682–0.777)***	Poor outcome: 4.85 (3.4–7.41), good outcome: 3.1 (2.01–4.43)***	1.059(0.904–1.241)**	9
Kim et al.	2022	Turkey	RCS	123	66.5 ± 12^a^	On admission	Full blood	EVT+IVT	mRS > 2 at 3 months	3 months	0.673(0.552–0.793)***	Poor outcome: 2029 (1217–2771), good outcome: 1172 (680–2145)**	2.28(1.06–4.88)**	0.717(0.595–0.838)***	Poor outcome: 9.7 (5.6–17.3), good outcome: 5.8 (3.1–9.0)***	–	9
Huang et al.	2023	China	RCS	234	69 (57–78)	On admission	Full blood	No surgery intervention	mRS > 2 at 3 months	3 months	0.678(0.608–0.748)***	–	2.350(1.149–4.803)**	–	–	–	8
Ma et al.	2023	China	RCS	190	70.389 ± 11.675^a^	On admission	Routine blood	EVT	mRS > 2 at 3 months	3 months	0.715(0.546–0.826)*	Poor outcome: 636.842 (461.159–1088.124), good outcome: 413.571 (264.923–654.741)***	1.003(1.001–1.005)**	–	Poor outcome: 3.509(2.838–7.237), good outcome: 2.159 (1.463–3.566)***	–	9
Li et al.	2025	China	RCS	381	LAA: 69.5 (58.8–78.0); SVO: 65.0 (54.0–74.8); CE: 75.5 (66.0–80.0); SOE: 68.0 (53.0–75.0); SUE: 63.5 (51.8–72.3)	Pre-thrombolysis	Routine blood	IVT	mRS > 2 at 3 months	3 months	–	–	–	0.701(0.646–0.755)***	Poor outcome: 4.0 (2.7–6.3), good outcome: 2.4 (1.8–3.7)***	1.261(1.148–1.384)***	9
Oh SW et al.	2020	Korea	RCS	411	69.9 ± 13.4^a^	On admission	Routine blood	EVT	mRS > 2 at 3 months	3 months	–	–	–	0.667(0.614–0.719)***	–	1.58(1.04–2.12)*	9
Deng et al.	2025	China	SC-RCS	151	74.28 ± 8.84^a^	Pre-thrombolysis	Routine blood	IVT	mRS > 2 at 3 months	3 months	–	–	–	0.711(0.622–0.800)***	Poor outcome: 3.83 (2.42–4.63), good outcome: 2.42(1.57–3.37)***	1.672(1.056–2.647)*	9
Chen et al.	2024	China	SC-RCS	303	67.30 ± 11.89^a^	On admission	Routine blood	EVT+IVT	mRS > 2 at 3 months	3 months	0.694(0.626–0.763)***	Poor outcome: 847.39 (531.30–1375.46), good outcome: 529.74 (373.78–841.03)***	–	0.717(0.648–0.786)***	Poor outcome: 3.93 (2.83–6.01), good outcome: 2.62 (1.94–3.99)***	1.148(1.04–1.27)**	9
Chen et al.	2021	China	PCS	448	66.8 ± 12.2^a^	On admission	Routine blood	No surgery intervention	mRS > 2 at 3 months	3 months	–	–	–	0.776(0.727–0.825)***	Poor outcome: 4.3 (2.9–6.2), good outcome: 2.3 (1.8–3.2)***	1.51(1.27–1.80)***	9
Li et al.	2021	China	RCS	286	70.00 (63.00–77.00)	Pre-mechanical thrombectomy	Routine blood	EVT	mRS > 2 at 3 months	3 months	–	–	–	0.732(0.673–0.791)***	Poor outcome: 5.28 (3.63–8.02), good outcome: 3.44 (2.63–4.63)***	1.957(1.382–2.770)***	9
Han et al.	2023	China	RCS	100	63.11 ± 7.45^a^	On admission	Routine blood	IVT	mRS > 2 at 3 months	3 months	–	–	–	0.811(0.712–0.911)*	–	1.185(1.078–3.012)**	9
Prandin G et al.	2025	United Kingdom, Italy, United States	RCS	970	69 (56–78)	On admission	Routine blood	MT+IVT	mRS > 2 at 3 months	3 months (±14 days)	–	Poor outcome: 1118.81 (569.75–1946.50), good outcome: 829.26 (424.34–1665.52)***	1.001(1.001–1.002)*	–	Poor outcome: 5.00 (2.67–9.33), good outcome: 4.06 (2.00–7.38)***	1.09(1.01–1.17)*	9

*Notes:* Values are presented as median (interquartile range) unless otherwise indicated. EVT; endovascular therapy; IVT: intravenous thrombolysis; NOS: Newcastle-Ottawa Scale; PCS: prospective cohort study; RCS, retrospective cohort study; SC-RCS: single-centre retrospective cohort study.

^a^Data presented as mean ± standard deviation. ‘–’ indicates data not extractable or not reported in the original study.

*p < .05,**p < .01,***p < .001.

### 3.2. Overall predictive performance of SII and NLR

**Figure 2.**
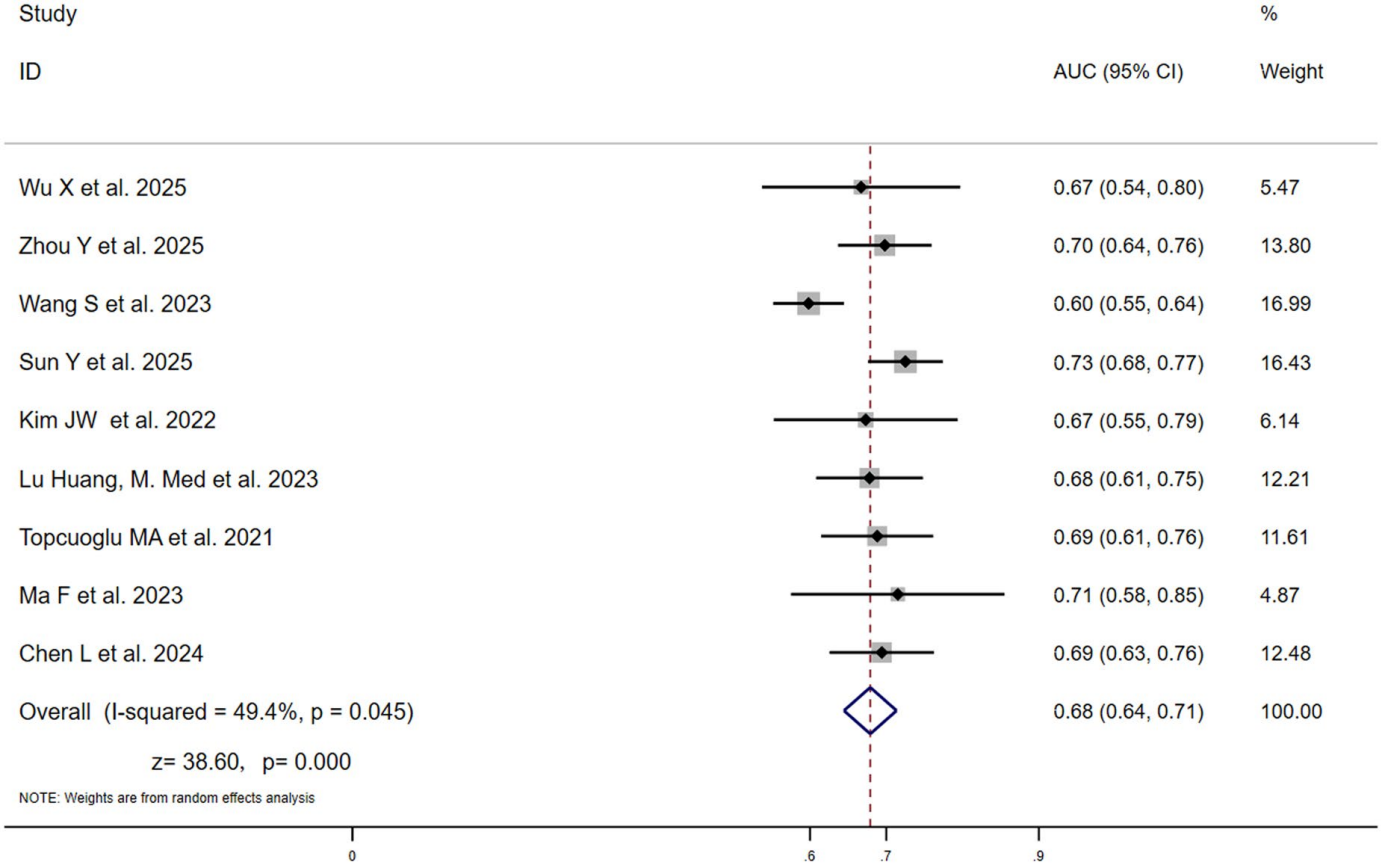
Forest plot of AUC for the predictive value of SII for three-month poor functional outcome (mRS > 2) in ischaemic stroke patients.

**Figure 3.**
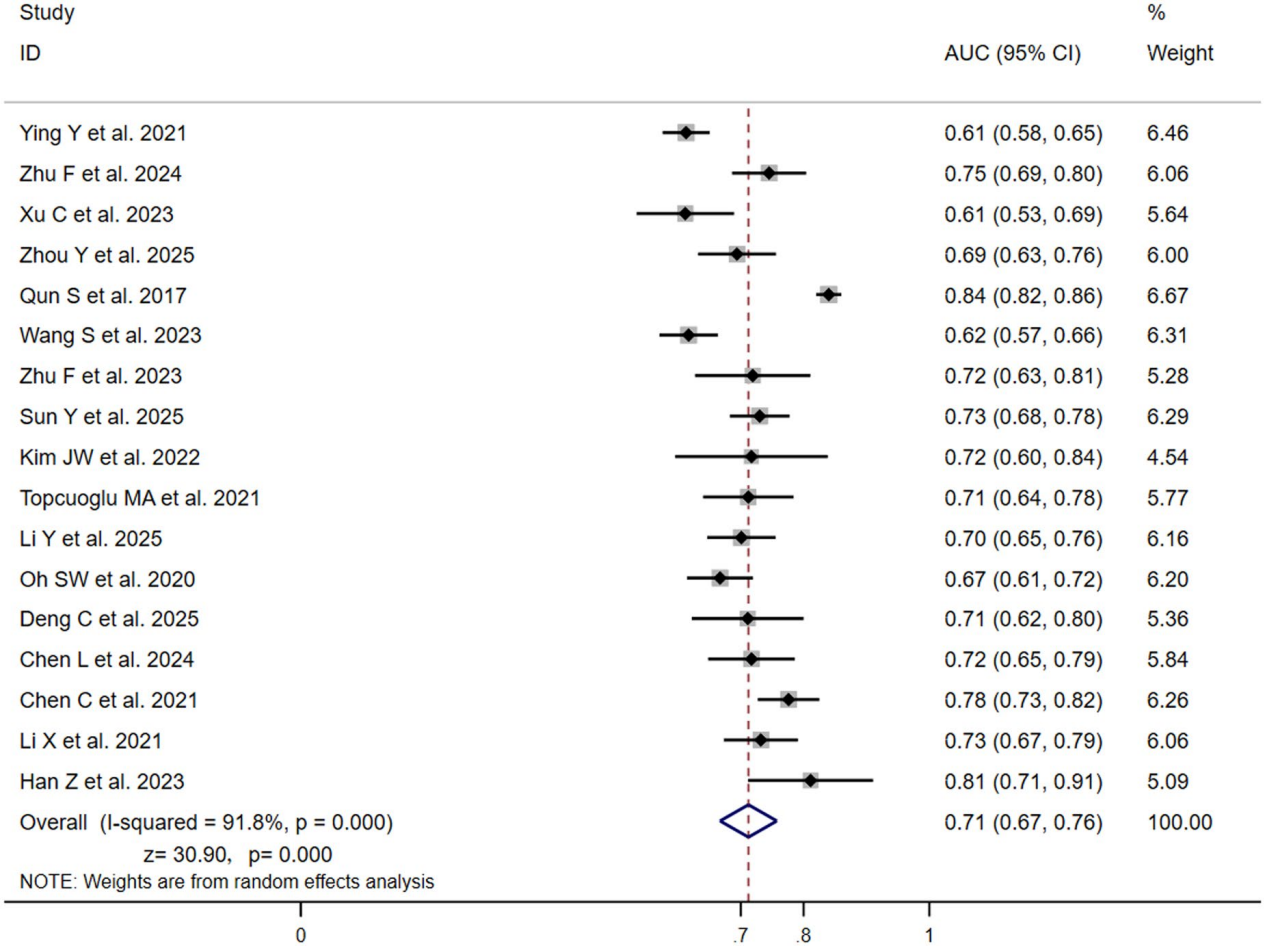
Forest plot of AUC for the predictive value of NLR for three-month poor functional outcome (mRS > 2) in ischaemic stroke patients.

Table 2 summarizes key findings. For diagnostic performance comparison, nine studies evaluated SII’s predictive value for poor three-month outcome (mRS >2) (Figure 2). Individual study AUCs ranged from 0.60 (95% CI: 0.55–0.64) to 0.73 (95% CI: 0.68–0.77), with Sun et al. [22] reporting the highest AUC (0.73) and Wang et al. [24] the lowest (0.60). Weight analysis indicated Wang et al. [24] and Sun et al. [22] contributed the highest weights (16.99% and 16.43% respectively), substantially influencing pooled results. Moderate heterogeneity (I^2^=49.4%, *p* = .045) warranted random-effects modelling, yielding pooled AUC = 0.68 (95% CI: 0.64–0.71; *z* = 38.60, *p* < .001), indicating moderate diagnostic value. Seventeen studies assessed NLR (Figure 3), showing wider AUC variability (0.61 [95% CI: 0.53–0.69] to 0.84 [95% CI: 0.82–0.86]), with Qun et al. [21] reporting maximum AUC (0.84) and Xu et al. [26] minimum (0.61). Qun et al. [21] carried the highest weight (6.67%), followed by Ying et al. [27] (6.46%). Significant heterogeneity (I^2^=91.8%, *p* < .001) necessitated random-effects modelling, yielding NLR’s pooled AUC = 0.71 (95% CI: 0.67–0.76; *z* = 30.90, *p* < .001), suggesting a slightly higher diagnostic performance for NLR versus SII (0.71 vs 0.68), though this difference was not statistically significant. Both values indicate a moderate level of predictive accuracy.

**Table 2.** Meta-analysis and subgroup analysis of clinical outcomes and predictive values.

Index	Items	Studies, *n*	Effect size (95% CI)	*p* Value	Heterogeneity (I², P for Cochran Q)
SII	Predictive value for poor outcome (AUC)				
	Pooled	9	0.68 (0.64, 0.71)	*p* < .00001	I²=49.4%, *p* = .045
	China	7	0.68 (0.64, 0.72)	*p* < .00001	I²=61.8%, *p* = .016
	Non-China	2	0.68 (0.62, 0.75)	*p* < .00001	I²=0.00%, *p* = .835
	IVT, EVT, or MT	7	0.68 (0.64, 0.72)	*p* < .00001	I²=62.0%, *p* = .015
	No Surgery Intervention	2	0.68 (0.61, 0.74)	*p* < .00001	I²=0.00%, *p* = .884
	Association with poor outcome (OR^a^)				
	Pooled	7	1.00 (1.00, 1.00)	*p* = .384	I²=86.6%, *p* = .000
	Difference in levels (SMD^b^)				
	Poor outcome/Good outcome	9	0.72 (0.48, 0.96)	*p* < .00001	I²=88.8%, *p* = .000
NLR	Predictive value for poor outcome (AUC)				
	Pooled	17	0.71 (0.67, 0.76)	*p* < .00001	I²=91.8%, *p* = .000
	China	14	0.72 (0.66, 0.77)	*p* < .00001	I²=93.0%, *p* = .000
	Non-China	3	0.69 (0.65, 0.73)	*p* < .00001	I²=0.00%, *p* = .534
	IVT, EVT, or MT	13	0.69 (0.66, 0.72)	*p* < .00001	I²=69.8%, *p* = .000
	No Surgery Intervention	4	0.78 (0.72, 0.84)	*p* < .00001	I²=83.1%, *p* = .000
	Association with poor outcome (OR^a^)				
	Pooled	14	1.26 (1.17, 1.37)	*p* < .00001	I²=75.5%, *p* = .000
	Difference in levels (SMD^b^)				
	Poor outcome/good outcome	15	0.69 (0.52, 0.87)	*p* < .00001	I²=88.8%, *p* = .000

*Notes:* Values are presented as pooled estimate (95% CI). AUC: area under the curve; CI: confidence interval; IVT: intravenous thrombolysis; EVT: endovascular therapy; MT: mechanical thrombectomy; OR: odds ratio; SMD: standardized mean difference.

^a^OR per unit increase in the biomarker.

^b^SMD comparing biomarker levels between poor (mRS >2) and good (mRS ≤2) outcome groups.

**Figure 4.**
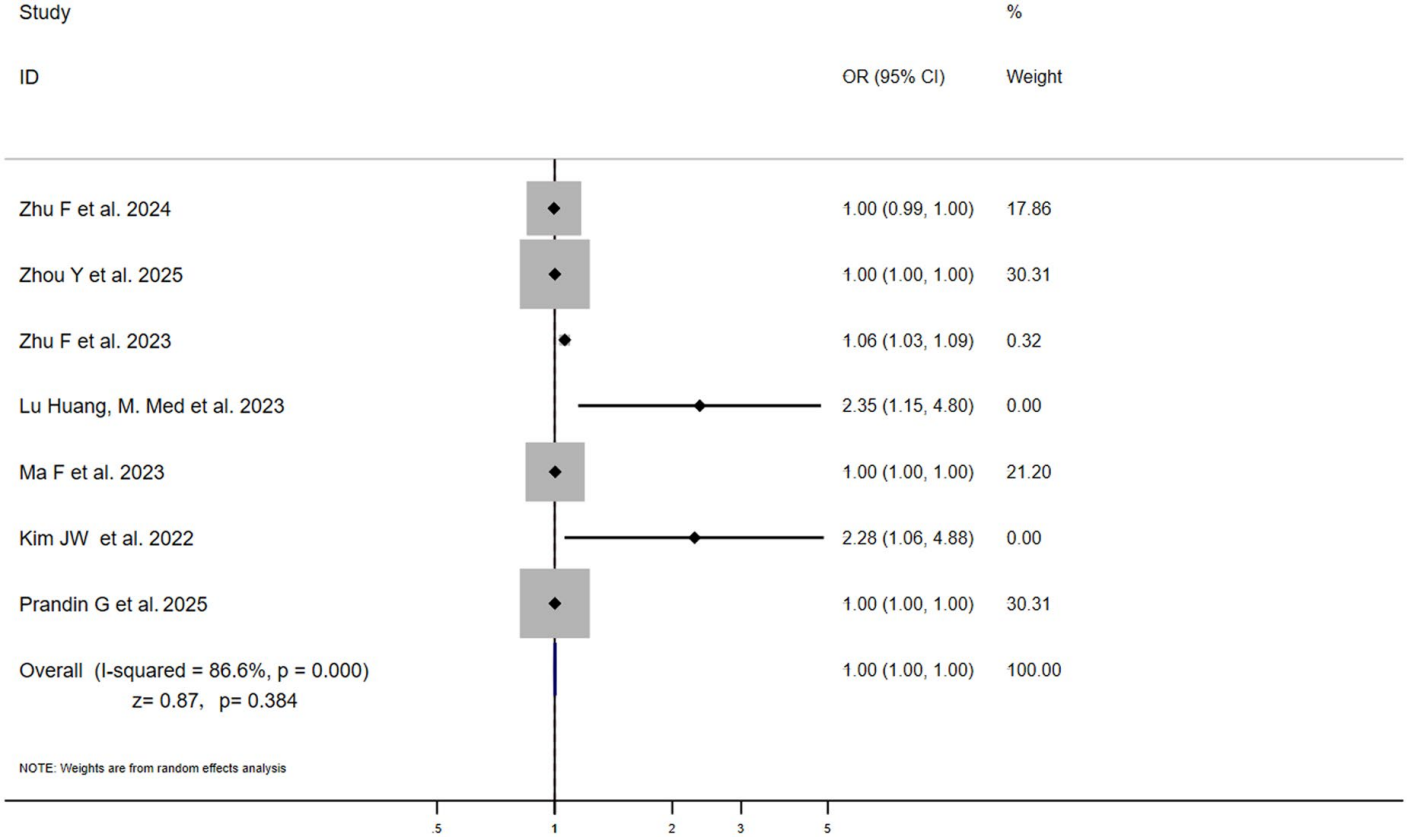
Forest plot of multivariable-adjusted OR for association between SII and three-month poor functional outcome (mRS > 2) in ischaemic stroke patients.

**Figure 5.**
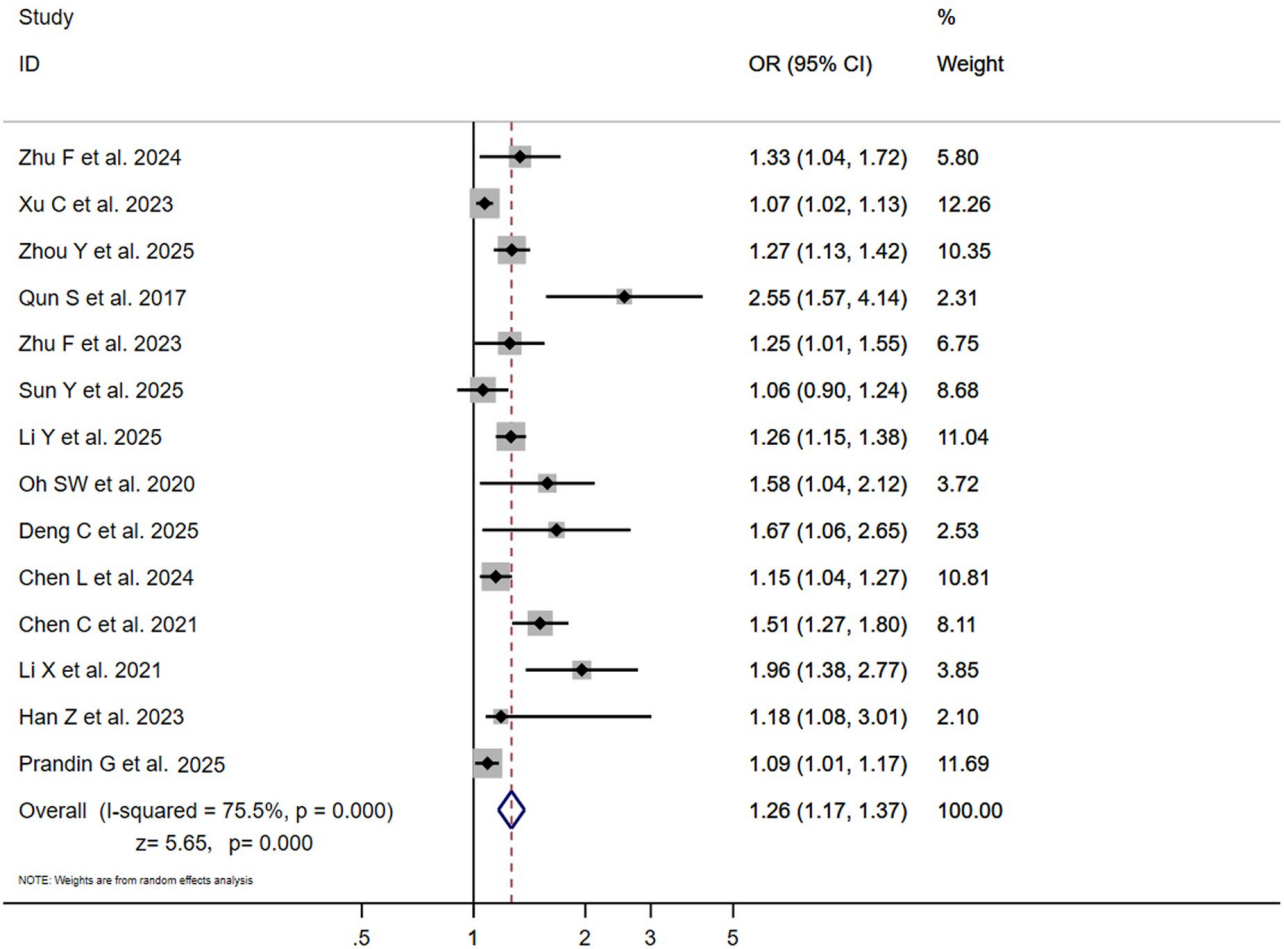
Forest plot of multivariable-adjusted OR for association between NLR and three-month poor functional outcome (mRS > 2) in ischaemic stroke patients.

Seven studies reported multivariable-adjusted ORs for SII and poor outcome (Figure 4). ORs varied substantially (1.00 [95% CI: 0.99–1.00] to 2.35 [95% CI: 1.15–4.80]), with Huang [8] reporting the highest OR (2.35) and Zhu et al. [30], Zhou et al. [28], Ma et al. [19], Prandin et al. [31] the lowest (1.00). High heterogeneity (I^2^=86.6%, *p* = .000) led to pooled OR = 1.00 (95% CI:1.00–1.00; *z* = 0.87, *p* = .384),indicating non-significant association. Fourteen studies reported NLR-adjusted ORs (Figure 5), ranging from 1.06 (95% CI: 0.90–1.24) to 2.55 (95% CI:1.57–4.14). Qun et al. [21] reported the highest OR (2.55), while Sun et al. [22] reported the lowest (1.06) with 95% CI crossing 1, indicating non-significance in that study. Xu et al., Prandin et al. and Li et al. [18,26,31] exerted greatest influence (weights:12.26%,11.69%,11.04%). High heterogeneity (I^2^=75.5%, *p* < .001) yielded pooled OR = 1.26 (95% CI: 1.17–1.37; *z* = 5.65, *p* < .001), indicating each unit NLR increase conferred 26% higher risk of poor outcome. This striking discrepancy suggests that while both markers are elevated in patients with poor outcomes, only NLR demonstrates an independent association with the risk of a poor prognosis after adjusting for potential confounders.

**Figure 6.**
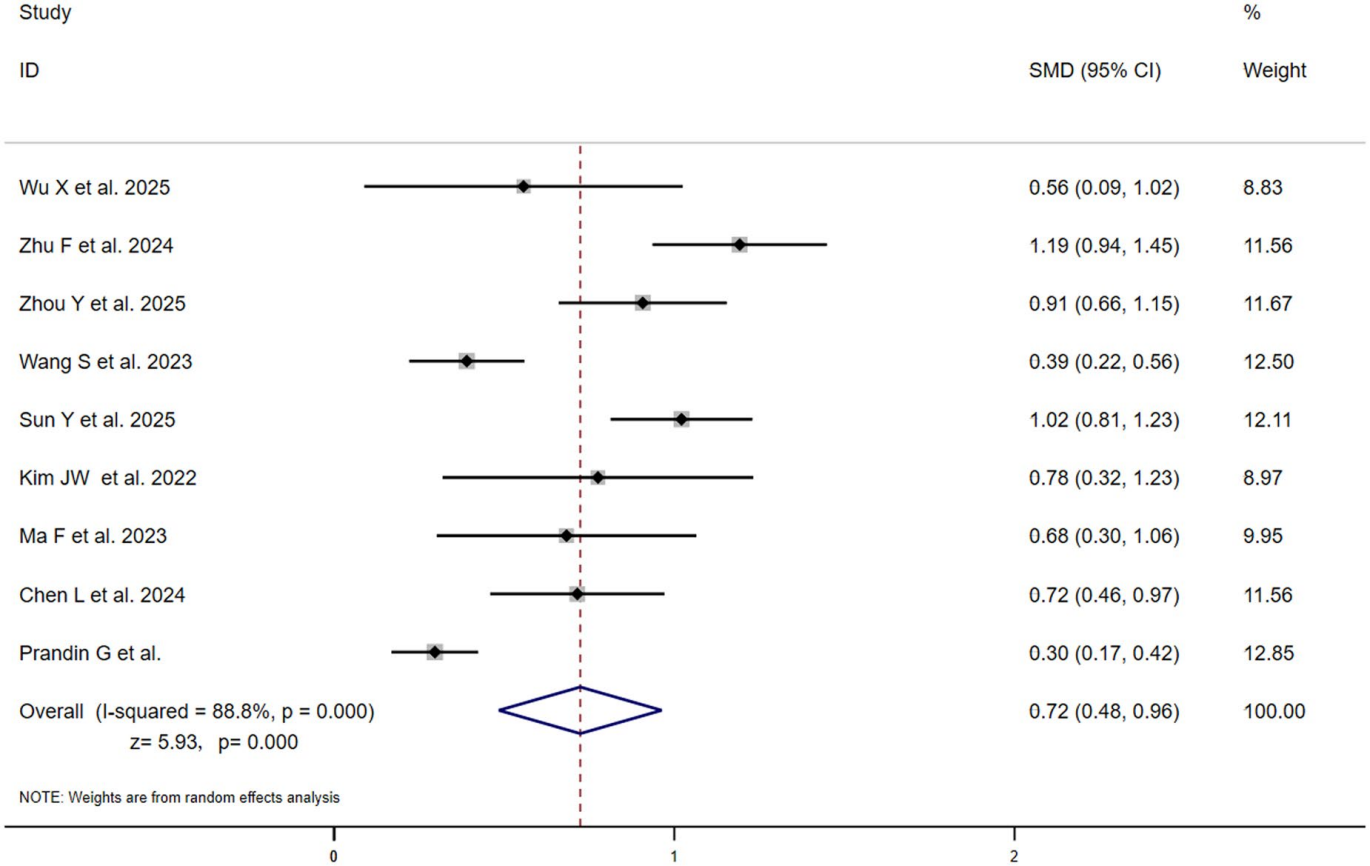
Forest plot of SMD for association between SII and poor outcome vs. good outcome in ischaemic stroke patients.

**Figure 7.**
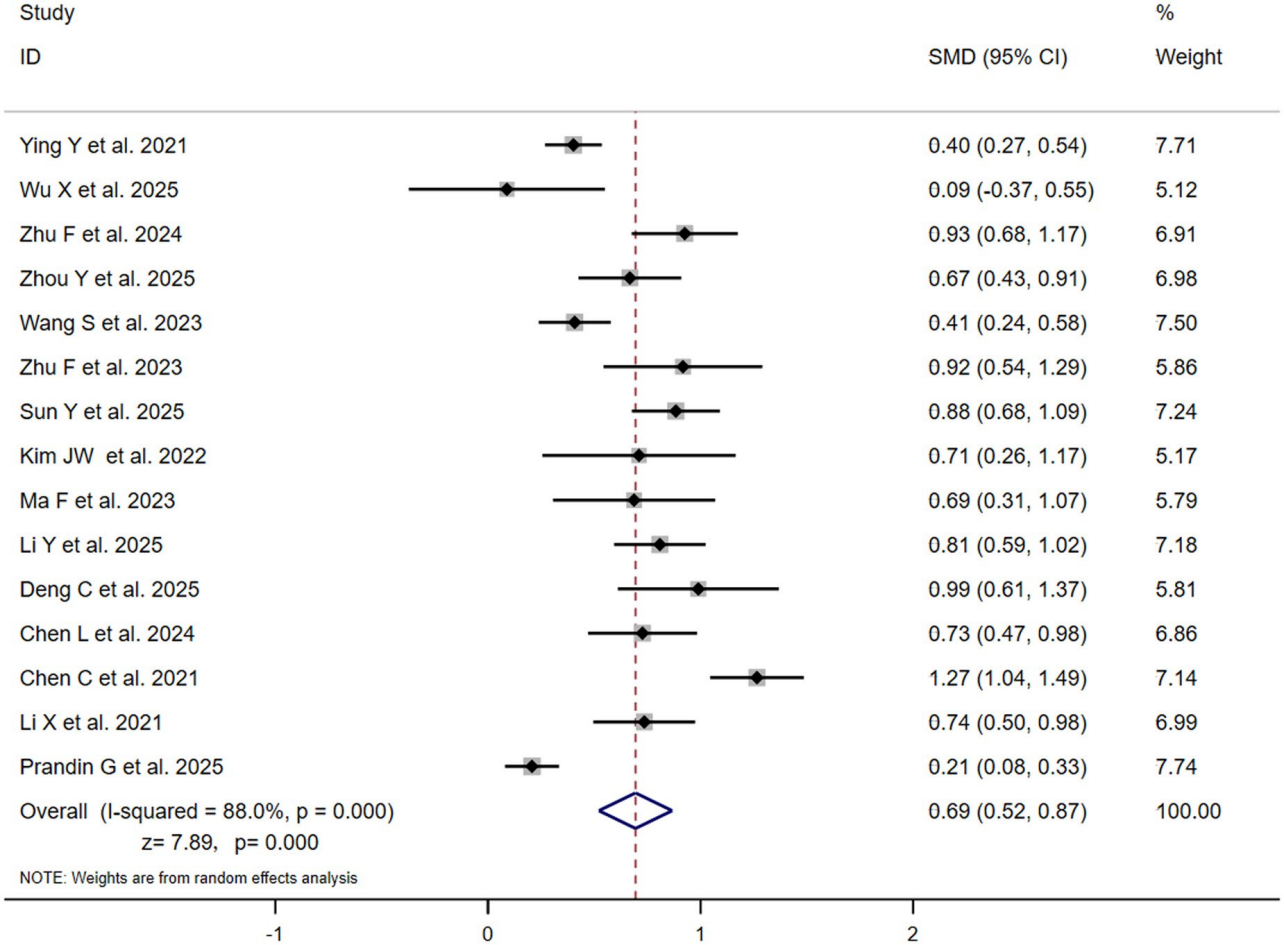
Forest plot of SMD for association between NLR and poor outcome vs. good outcome in ischaemic stroke patients.

Nine studies compared SMD between outcome groups for SII (Figure 6). SMDs ranged from 0.30 (95% CI: 0.17–0.42) to 1.19 (95% CI: 0.94–1.45), with Zhu et al. [30] reporting maximum SMD (1.19) and Prandin et al. [31] minimum (0.30). Prandin et al. [31] carried highest weight (12.85%), followed by Wang et al. [24] (12.50%). High heterogeneity (I^2^=88.8%, *p* = .000) produced pooled SMD = 0.72 (95% CI:0.48–0.96;*z* = 5.93, *p* = .000), indicating significantly elevated SII in poor-outcome group with moderate effect size (SMD > 0.5). Fifteen studies provided NLR data (Figure 7), with SMDs ranging from 0.09 (95% CI:−0.37–0.55) to 1.27 (95% CI: 1.04–1.49). Chen et al. [13] reported maximum SMD (1.27), while Wu et al. [25] reported minimum (0.09) with 95% CI crossing zero. Prandin et al., Ying et al., and Li et al. [18,27,31] contributed highest weights (7.74%,7.71%,7.18%). High heterogeneity (I^2^=88.0%, *p* = .000) yielded pooled SMD = 0.69 (95% CI:0.52–0.87; *z* = 7.89, *p* = .000), indicating significantly higher NLR in the poor-outcome group with a moderate effect size, marginally lower than SII’s SMD (0.72 vs 0.69) without statistical significance. The slightly higher SMD for SII, despite its non-significant risk association, indicates a more pronounced absolute difference in SII values between outcome groups, which may be influenced by unadjusted confounding factors.

### 3.3. Subgroup analyses and sources of heterogeneity

Subgroup analyses of AUC were conducted by treatment (IVT, EVT, or MT vs. none) and country (China vs. non-China). NLR exhibited higher predictive performance in non-intervention groups (AUC = 0.78, 95% CI: 0.72–0.84) than in intervention groups (AUC = 0.69, 95% CI: 0.66–0.72) (Supplementary Figure 1B), suggesting that reperfusion may attenuate NLR’s prognostic value. In contrast, SII showed consistent AUC values (0.68) across treatment groups (Supplementary Figure 2B), indicating greater stability as a treatment-agnostic biomarker. When stratified by country, NLR demonstrated marginally higher AUC in Chinese populations (0.72 vs. 0.69) but with substantial heterogeneity (I^2^ = 93.0%; Supplementary Figure 1A), while SII maintained an AUC of 0.68 in both subgroups with better consistency (Supplementary Figure 2A). Additional subgroup analyses (e.g. by study design or stroke subtype) were predefined but not feasible due to critical data gaps: only 2 of 17 NLR-related studies were prospective (insufficient statistical power for subgroup stratification), and 16 studies did not report detailed stroke subtype information. Significant residual heterogeneity persisted for NLR despite sensitivity analyses, which is likely multifactorial – attributed to unmeasured confounders (e.g. pre-stroke inflammatory comorbidities), non-standardized sampling timelines for biomarker measurement, and unreported clinical details (e.g. variations in post-stroke medication use). For SII, heterogeneity was relatively milder and more consistent across subgroups, reflecting its greater stability as a prognostic marker. Nevertheless, the differential influence of treatment and geography on NLR versus the stability of SII remains clinically informative.

### 3.4. Risk of bias and publication bias assessment

Methodological quality was assessed using NOS. All studies scored ≥6 (median= 7, range: 6–9), indicating high quality. Detailed ratings are in Supplementary Table 2. Publication bias was evaluated using Begg’s and Egger’s tests (*p* < .05 significance; Supplementary Table 3). For SII, no significant bias was found across outcomes: AUC (Begg’s z = −1.04, *p* = .297; Egger’s *p* = .568), OR (Begg’s *z* = 1.05, *p* = .293; Egger’s *p* = .258), and SMD (Begg’s *z* = 0.10, *p* = .917; Egger’s *p* = .123). Subgroup analyses consistently showed no bias. For NLR, no significant bias was observed for AUC (Begg’s *z* = 0.95, *p* = .343) or SMD (Begg’s *z* = 0.10, *p* = .921; Egger’s *p* = .051), but Egger’s test indicated bias for AUC (*p* = .028), particularly in the China (*p* = .042) and no-intervention (*p* = .017) subgroups. For OR, Begg’s test showed no bias (*z* = 1.75, *p* = .080), but Egger’s test was significant (*p* = .001). It is critical to note that while the total number of NLR-related studies included in this meta-analysis is sufficient, Egger’s test for detecting publication bias may still have inherent limitations. For instance, the test performance could be affected by individual studies with extreme effect sizes, which limits its ability to accurately identify true publication bias. Thus, while these results suggest potential bias, they should be interpreted with caution. We also acknowledge that publication bias might overestimate the predictive efficacy of NLR. To address the impact of high heterogeneity on pooled estimates, we performed leave-one-out sensitivity analysis for NLR’s AUC, OR, and SMD. For NLR’s AUC, sequentially excluding each study and recalculating the pooled effect sizes showed the estimates ranged from approximately 0.67 to 0.76, with no substantial fluctuations and relatively consistent confidence intervals across exclusions, confirming the robustness of the pooled AUC finding. For NLR’s OR, the pooled estimates ranged from around 0.15 to 0.36 upon excluding each study, and the overall pattern of effect sizes remained stable without extreme deviations, indicating the pooled OR result is robust. For NLR’s SMD, the estimates varied between approximately 0.52 and 0.91 when each study was excluded in turn, with no significant shifts in effect sizes, verifying the robustness of the pooled SMD conclusion. Collectively, these analyses revealed no substantial fluctuations in effect sizes, confirming the robustness of our core findings. Future studies with more standardized designs (e.g. unified sampling timing and outcome definition) are needed to further verify the prognostic value of NLR. Supplementary Figures 3–14 provide publication bias funnel plots and sensitivity analysis forest plots.

## 4. Discussion

The inflammatory response following acute ischaemic stroke critically influences neurorecovery by activating immune cells, releasing inflammatory mediators, and disrupting the blood–brain barrier – a core mechanism affecting functional restoration [32]. Monitoring dynamic changes in systemic inflammatory biomarkers is crucial for improving prognostic accuracy and guiding immunomodulatory therapies. NLR and SII integrate peripheral immune cell counts, reflecting both innate immunity (*via* neutrophils) and adaptive immune status (*via* lymphocytes), while also capturing platelet-mediated thromboinflammatory crosstalk [33,34]. Unlike single inflammatory markers such as C-reactive protein, NLR and SII can be calculated from routine blood tests, offering greater accessibility, cost-effectiveness, and practicality in primary and emergency care settings; more importantly, by capturing systemic immunoinflammatory dynamics, these two indices provide incremental value beyond traditional prognostic tools (e.g. the National Institutes of Health Stroke Scale [NIHSS] and Alberta Stroke Program Early Computed Tomography Score [ASPECTS]) [35], facilitating risk stratification in resource-limited environments and thereby promoting more efficient clinical application. However, their comparative prognostic value in ischaemic stroke remains uncertain, and the mechanisms underlying their association with adverse outcomes have not been fully elucidated. Therefore, they represent promising exploratory biomarkers – currently suitable for use as supplements to existing indicators in early triage. NLR directly captures the post-ischaemic ‘pro-inflammatory/anti-inflammatory imbalance’ *via* neutrophil–lymphocyte dynamics [36]. Shortly after ischaemic injury, neutrophils infiltrate the affected tissue and release reactive oxygen species and matrix metalloproteinases (MMPs), contributing to blood–brain barrier disruption and neuronal necrosis. Ruhnau et al. identified neutrophils as the first immune cells to invade after cerebral ischaemia, promoting thrombosis and neuroinflammation through protease and neutrophil extracellular trap (NET) release [37]. Schuhmann et al. described time-dependent immune cell migration, with neutrophils and macrophages accumulating rapidly and initiating inflammatory cascades [38]. Yang et al. highlighted the central role of MMPs in regulating blood-brain barrier permeability in ischaemic stroke [39]; In contrast, lymphocytes – particularly Treg cells – suppress excessive inflammation through anti-inflammatory cytokines such as IL-10 and TGF-β, facilitating tissue repair. Schuhmann MK et al. observed elevated pro-inflammatory cytokines (TNF-α, IL-6), expanded infarct volumes, and increased vascular permeability in Treg-deficient mice, exacerbating secondary injury [38]. Ito M et al. demonstrated that post-ischaemic Treg accumulation inhibits astrocytic activation *via* amphiregulin, promoting recovery [40]. Consistent with these mechanisms, an elevated NLR reflects pro-inflammatory dominance, aligning with our significant association between NLR and poor outcome (OR = 1.26). Furthermore, SII incorporates platelet counts and may more comprehensively capture inflammation–thrombosis crosstalk. Platelets facilitate thrombosis and form platelet–neutrophil aggregates (PNAs) *via* P-selectin binding to PSGL-1, enhancing neutrophil activation, migration, and local inflammation through ROS and pro-inflammatory cytokines (e.g. IL-6, TNF-α) [41]. PNAs also promote neutrophil extracellular trap (NET) release, leading to microvascular occlusion and hypoxia [42]. This cyclic inflammation-thrombosis interaction may explain SII’s slightly greater between-group effect size (SMD = 0.72 vs. 0.69 for NLR), though its lack of independent association (OR = 1.00) suggests more complex regulatory mechanisms. This discrepancy may stem from the fundamental differences in what SMD and OR measure, as well as the unique biological properties of each biomarker. The SMD quantifies the absolute magnitude of between-group differences in biomarker levels without accounting for confounding factors [43] – SII’s larger SMD reflects its characteristic of integrating platelet counts, capturing both inflammatory and thrombotic pathways that are concurrently elevated in patients with higher baseline vascular burden (e.g. complicated with hypertension, acute thrombus burden) and poor prognosis [44]. In contrast, the OR assesses independent prognostic associations after adjusting for confounding factors such as age, stroke severity, and treatment modality. SII’s non-significant OR indicates that its elevated levels in the poor prognosis group are largely explained by these confounders, whereas NLR – focusing solely on the neutrophil-lymphocyte balance, a direct surrogate for post-ischaemic inflammatory dysregulation [45] – retains a robust, confounder-independent association with prognosis. This aligns with NLR’s role in inflammation-driven neurological recovery (as demonstrated in our subgroup analysis, modulated by reperfusion), while SII functions as a ‘treatment-agnostic’ marker of baseline systemic stress (inflammation + thrombosis) [44].

Prior studies on NLR and SII in ischaemic stroke align with and extend our results. Lu et al. established NLR as an independent prognostic marker (AUCs 0.65–0.75), consistent with our AUC of 0.71 [46]; Huang reported SII significantly predicted poor outcome (OR = 2.35, 95% CI: 1.15–4.80; AUC = 0.678), albeit in a single-centre study [8]. Xiao et al. found SII prognostic in intracerebral haemorrhage (AUC = 0.662), though limited by small sample size (*n* = 271) [47]. Our meta-analysis of 9 studies confirms SII’s moderate accuracy (AUC = 0.68). NLR performed better in non-intervention patients (AUC = 0.78), consistent with Wu et al., who reported higher AUC in non-recanalized (0.75) vs. recanalized patients (0.62) [48]. This pattern of NLR’s prognostic utility being modulated by intervention is further supported by a multicentre study of 970 patients with large vessel occlusion (LVO)-related acute ischaemic stroke undergoing mechanical thrombectomy (MT): Prandin et al. demonstrated that NLR – particularly the 24-h NLR – was significantly associated with 90-day unfavourable outcomes, with adjusted odds ratios (ORs) confirming its independent prognostic value; notably, 24-h dynamic NLR measurements offered greater clinical relevance than admission values and showed superior prognostic specificity in the MT population. In contrast, while the systemic immune-inflammation index (SII) was significantly elevated in the poor outcome group, its predictive performance was inferior to that of NLR, and its prognostic association became unstable after adjusting for clinical confounders [31]. These findings are highly consistent with those of our meta-analysis, which identified NLR (OR = 1.26) as an independent prognostic factor while SII showed no significant association with poor outcome risk, providing targeted clinical evidence for both the observed differences in prognostic value between the two markers and our analysis of NLR’s prognostic differences in the intervention subgroup. Reperfusion may reduce inflammation, diminishing NLR’s prognostic role. In contrast, SII remained consistent across treatments (AUC = 0.68), supporting its treatment-agnostic nature. Chu et al. found higher SII in poor-outcome patients (531.93 vs 397.23, *p* < .001), though not significant multivariately, suggesting thrombolysis may moderate its prediction [49]. These findings complement research on SII’s prognostic value post-thrombolysis.

Three key findings emerge from this analysis: First, NLR exhibited a slightly higher diagnostic accuracy than SII (AUC = 0.71 vs. 0.68), though this difference was not statistically significant – both demonstrated moderate performance, supporting their complementary use with established indicators such as NIHSS; second, NLR was significantly associated with poor outcome (OR = 1.26), while SII was not, indicating NLR’s stronger specificity for inflammation-driven prognosis; third, NLR performance varied by treatment and geography (non-intervention > intervention, Chinese > non-Chinese), whereas SII exhibited consistent accuracy across subgroups, supporting its use as a context-independent biomarker.

## 5. Limitations

This study has several limitations, among which the most important to emphasize is the significant constraint on its generalizability: among the 21 included studies, 17 (81%) were conducted in Chinese populations. This over-representation raises substantial concerns about extrapolating the findings to broader, multiethnic populations, primarily driven by three key factors: first, ethnic and genetic differences – such as polymorphisms in inflammation-related genes (e.g. TNF-α, IL-6) – which may alter the prognostic relevance of NLR and SII, as these genetic variations exhibit significant disparities between Asian and non-Asian populations [50]. Second, there are disparities in clinical practice patterns: differences exist in stroke treatment pathways, medication selection preferences, and post-stroke management protocols between China and Western countries, which may alter the correlation between NLR/SII and functional outcomes [51]. Third, there are differences in the spectrum of baseline comorbidities: the prevalence patterns of hypertension, diabetes mellitus, and dyslipidaemia in Chinese cohorts often exhibit uniqueness and differ from those in European and American populations, and these discrepancies may confound the association between inflammatory biomarkers and adverse outcomes [52]. Currently, data on SII in non-Asian populations remain scarce, precluding direct comparisons and further highlighting the uncertainty regarding the utility of SII in non-Chinese settings. Beyond generalizability, additional limitations include: first, the evidence base is primarily derived from retrospective observational studies, which are prone to biases arising from incomplete clinical data and unmeasured confounders. Second, significant heterogeneity was observed in key analyses (e.g. I^2^ = 91.8% for NLR AUC; I^2^ = 86.6% for SII OR; I^2^ = 88.8% for SII SMD), which can be attributed to several interrelated factors [1]: non-standardized sampling timelines for biomarker measurement (ranging from admission to 72 h post-stroke), with existing evidence indicating that NLR exhibits dynamic changes that may vary over time [2]; heterogeneity in treatment strategies across studies, as our subgroup analysis confirmed that recanalization therapy attenuates the prognostic utility of NLR; and [3] differences in study designs (retrospective vs. prospective) and outcome assessment protocols (e.g. subtle variations in mRS rater training). It is important to note that this study strictly standardized the core definition of adverse outcomes (mRS > 2) and systematically excluded all studies using alternative thresholds (e.g. mRS ≥ 2, mRS > 3, etc.), maximizing the consistency of outcome definition; while the aforementioned subtle differences in assessment may have a minor impact on inter-study comparability, the uniformity of the core threshold minimizes the risk of bias on the pooled results. Furthermore, several pre-specified subgroup analyses (e.g. stratification by stroke subtype) could not be performed because most original studies did not report detailed subtype information, limiting the robustness and interpretability of the results. Despite these limitations, this systematic review provides a critical comparative evaluation of SII and NLR in the prognostic assessment of ischaemic stroke, offering valuable insights for risk stratification in Chinese populations. To address the generalizability gap and reduce heterogeneity arising from variable sampling timelines, future studies should prioritize multi-centre prospective designs incorporating diverse ethnic groups to validate the prognostic performance of NLR and SII across varied genetic, clinical, and demographic contexts; additionally, these studies should standardize the sampling time for biomarkers and report precise time points to minimize this source of heterogeneity, enabling more rigorous subgroup analyses by sampling time.

## 6. Conclusion

In summary, our findings demonstrate that both NLR and SII serve as cost-effective, readily accessible inflammatory biomarkers for predicting poor three-month outcome (mRS > 2) in ischaemic stroke patients. NLR exhibited a slightly higher overall diagnostic accuracy than SII (AUC = 0.71 vs 0.68), though this difference was not statistically significant. It also demonstrated a significant association with poor outcome risk (OR = 1.26), suggesting greater clinical value in assessing ‘inflammation-driven prognosis’; conversely, while SII showed non-significant risk association, its diagnostic performance remained more stable across treatment modalities (surgical vs non-surgical) and geographical populations (Chinese vs non-Chinese), supporting its utility as a cross-context supplemental indicator. Between-group difference analysis confirmed both effectively discriminated poor from good outcome groups, achieving moderate effect sizes (SMD = 0.69 and 0.72, respectively).

As convenient routine tests, NLR and SII enable rapid identification of high-risk patients, particularly in primary care or emergency settings, informing prognosis stratification and therapeutic decisions. However, substantial heterogeneity (e.g. I^2^=91.8% for NLR AUC analysis, I^2^=86.8% for SII OR analysis) and NLR’s susceptibility to treatment modality/geographical influences necessitate further validation of their temporal dynamics (e.g. pre-/post-treatment fluctuations) for prognostic prediction. Future studies should implement standardized protocols (e.g. uniform sampling timing, refined stroke subtyping) and explore synergistic integration with conventional indicators (e.g. NIHSS scores) to enhance precision in ischaemic stroke prognosis assessment.

## Supplementary Material

Supplemental Material

## Data Availability

The data that support the findings of this study are available from the corresponding author upon reasonable request.
