# Supplementary figures and images for: Comparison of the predictive performance of systemic immune-inflammation index and neutrophil-to-lymphocyte ratio for three-month poor functional outcome in ischemic stroke: a systematic review and meta-analysis
Source: Ann Med. 2026 Jan 12;58(1):2612820. doi: 10.1080/07853890.2026.2612820 (PMC12802519; doi:10.1080/07853890.2026.2612820)

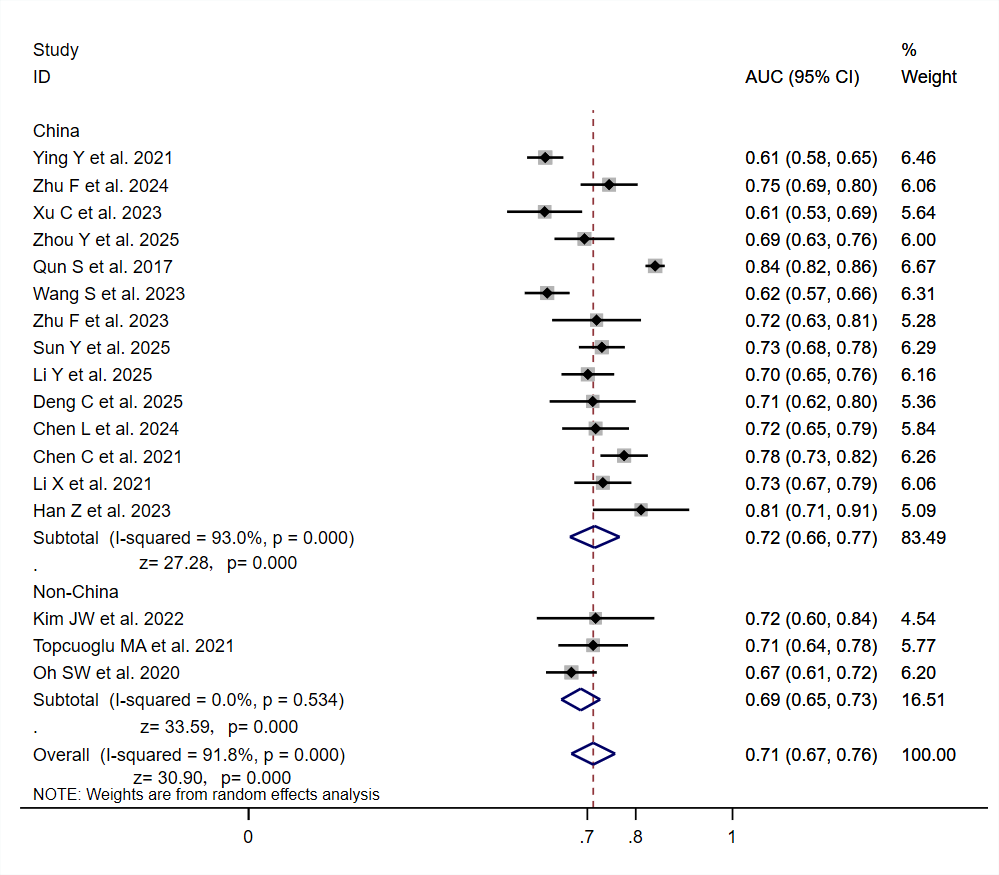

Supplement: Supplemental Material [file IANN_A_2612820_SM3028.zip › suppl_data/Supplementary Figure 1 A.tiff]

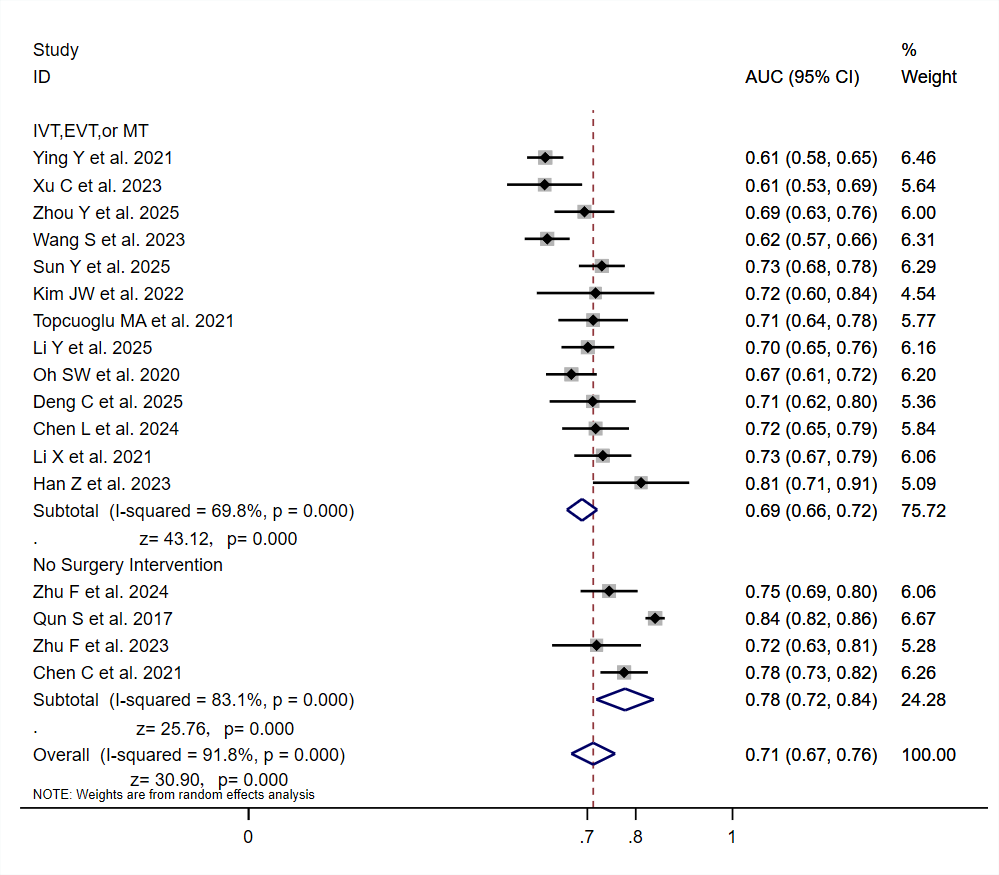

Supplement: Supplemental Material [file IANN_A_2612820_SM3028.zip › suppl_data/Supplementary Figure 1 B.tiff]

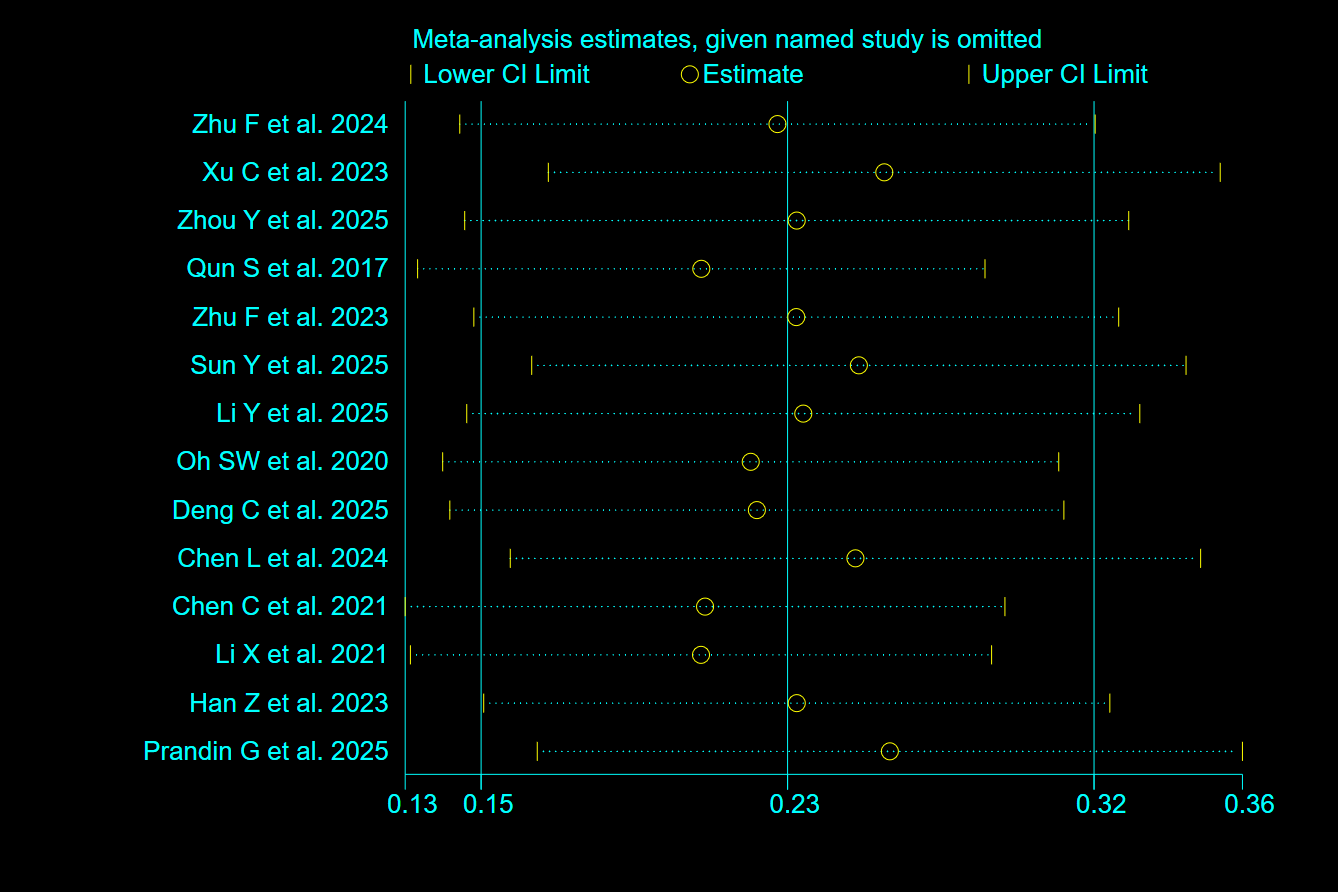

Supplement: Supplemental Material [file IANN_A_2612820_SM3028.zip › suppl_data/Supplementary Figure 10.tiff]

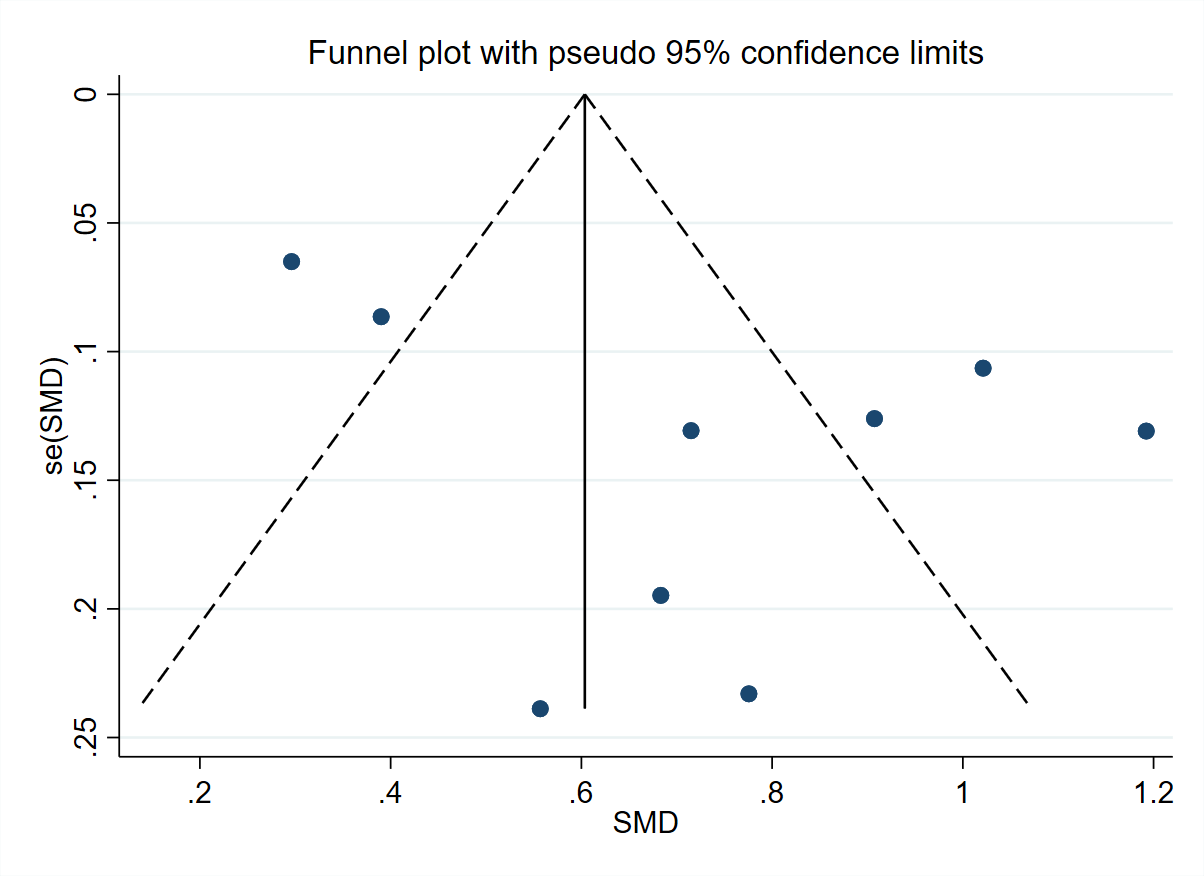

Supplement: Supplemental Material [file IANN_A_2612820_SM3028.zip › suppl_data/Supplementary Figure 11.tiff]

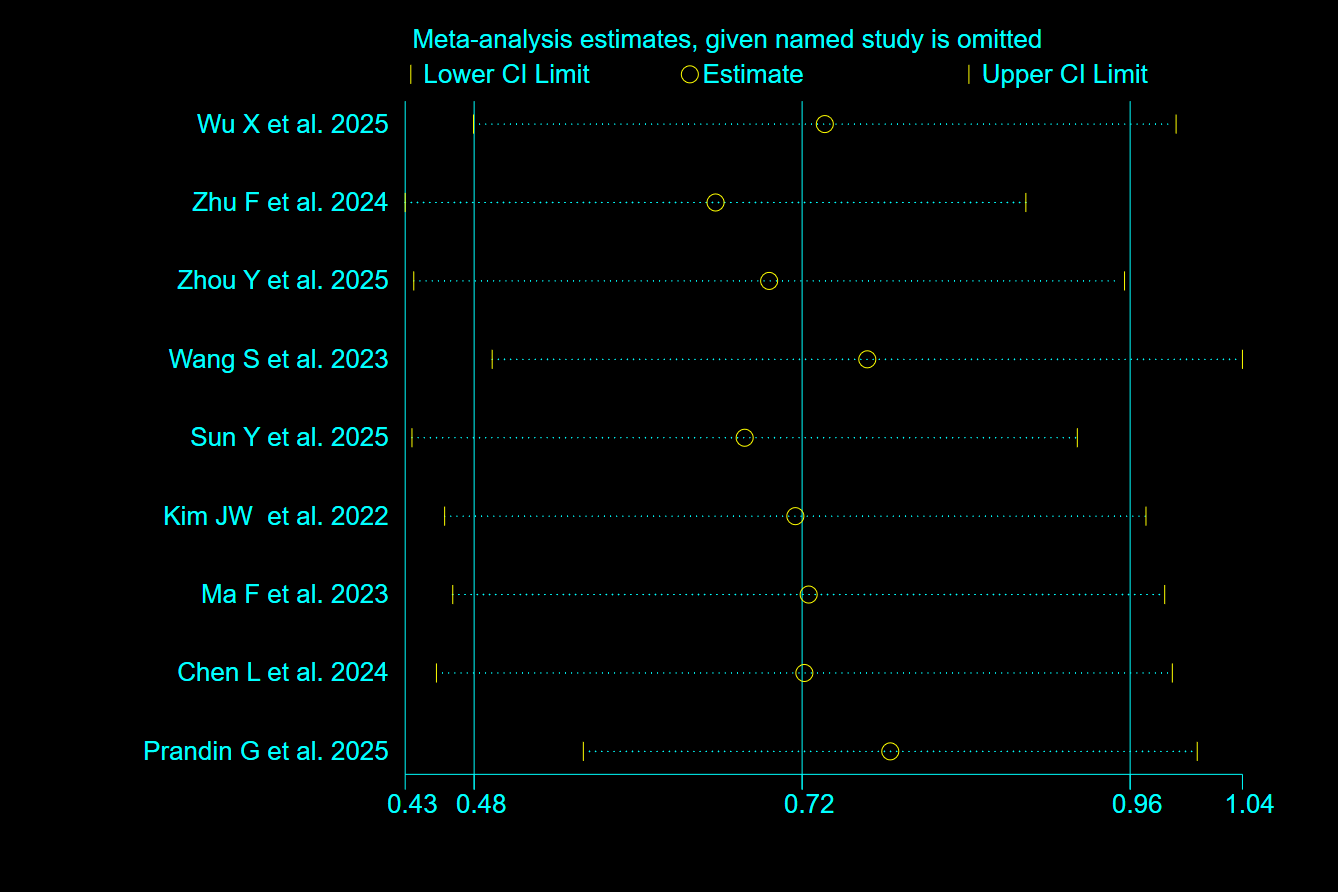

Supplement: Supplemental Material [file IANN_A_2612820_SM3028.zip › suppl_data/Supplementary Figure 12.tiff]

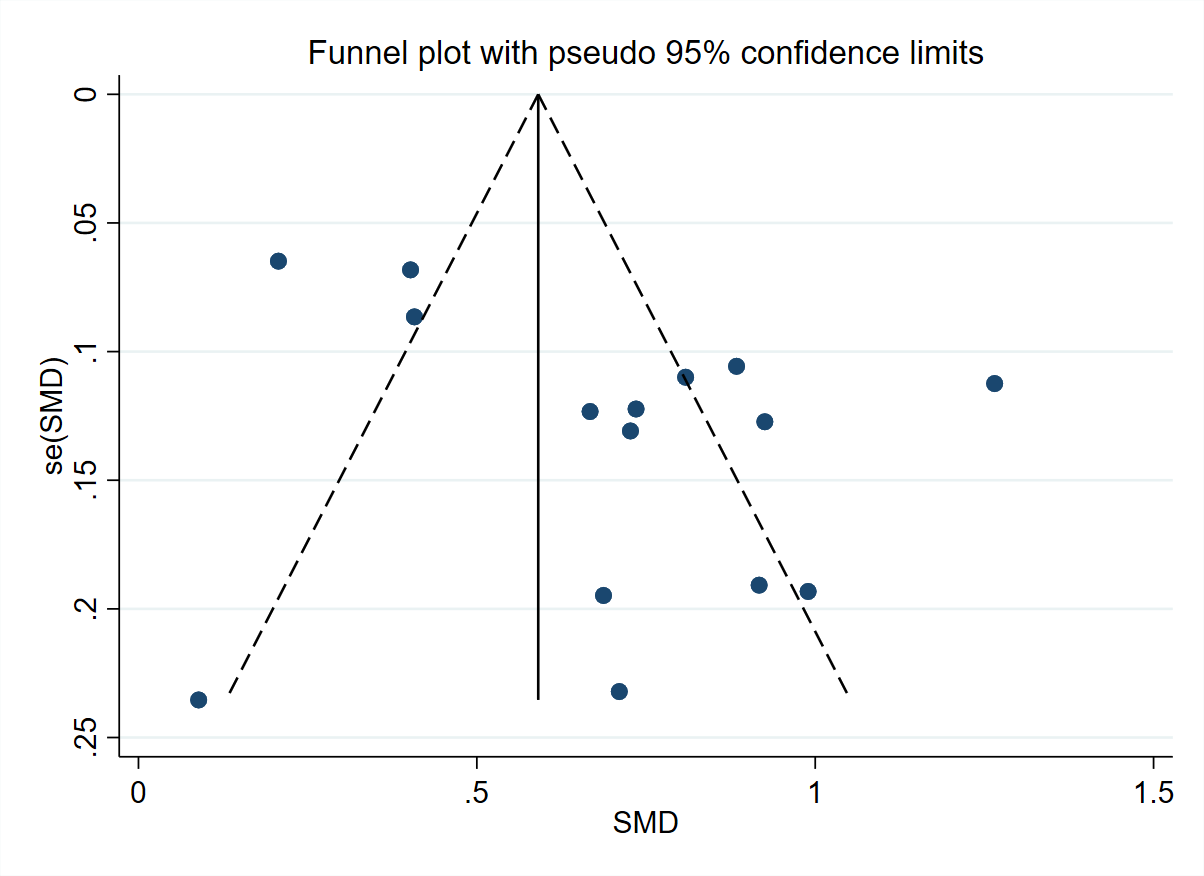

Supplement: Supplemental Material [file IANN_A_2612820_SM3028.zip › suppl_data/Supplementary Figure 13.tiff]

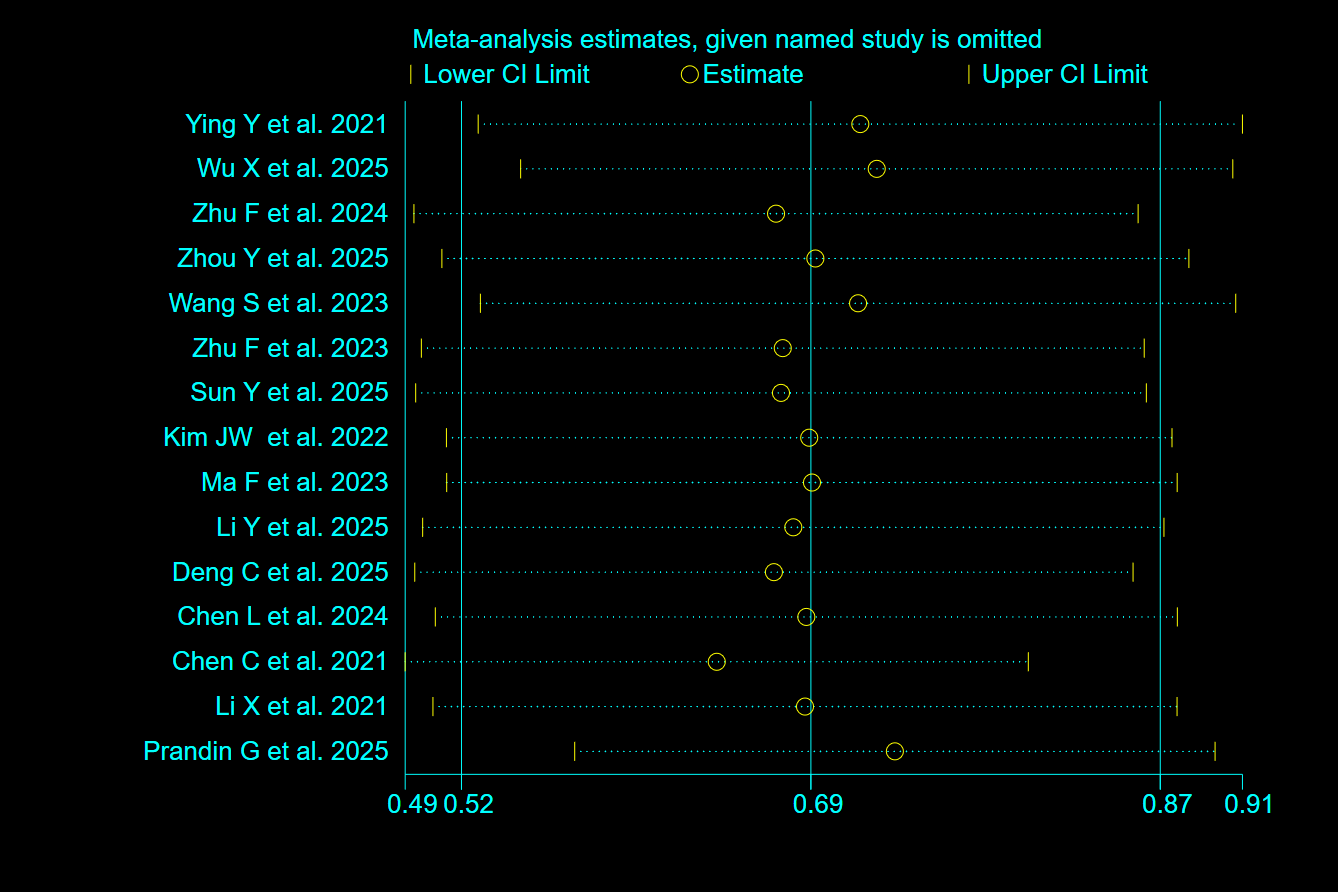

Supplement: Supplemental Material [file IANN_A_2612820_SM3028.zip › suppl_data/Supplementary Figure 14.tiff]

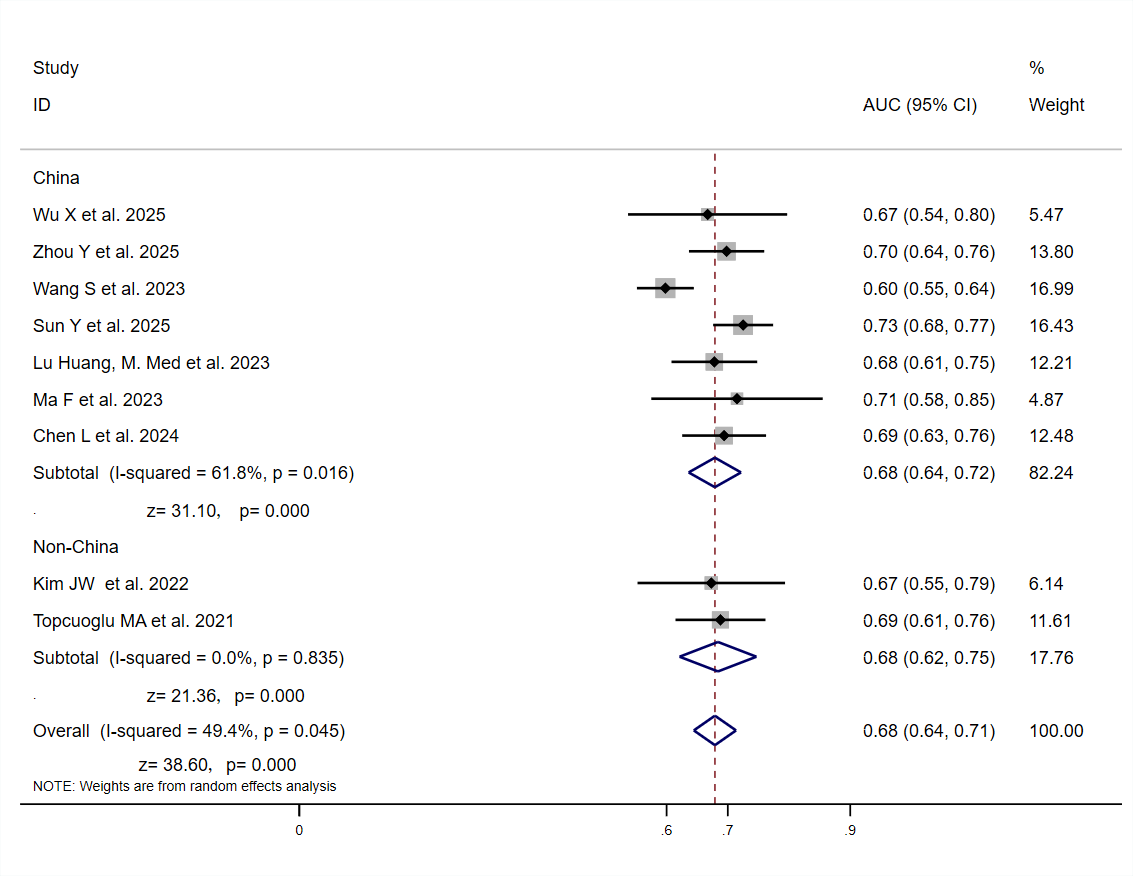

Supplement: Supplemental Material [file IANN_A_2612820_SM3028.zip › suppl_data/Supplementary Figure 2 A.tiff]

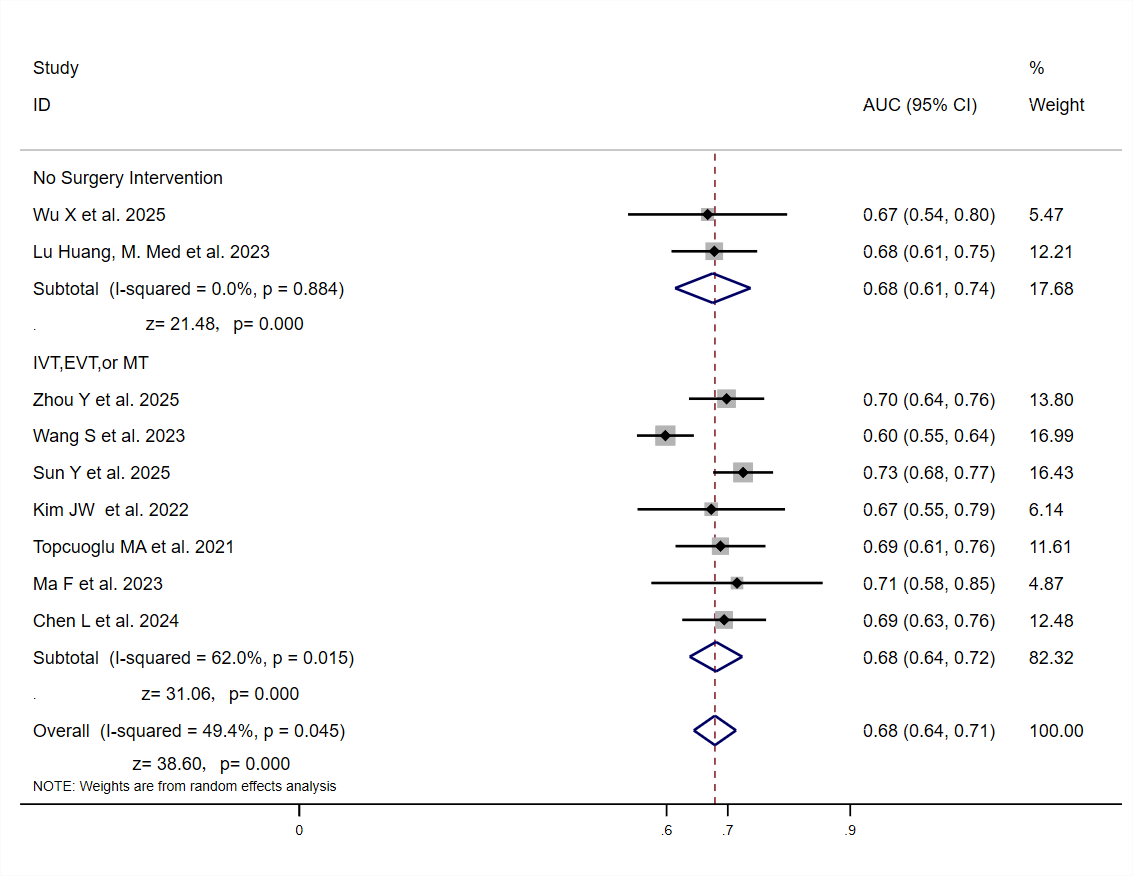

Supplement: Supplemental Material [file IANN_A_2612820_SM3028.zip › suppl_data/Supplementary Figure 2 B.tiff]

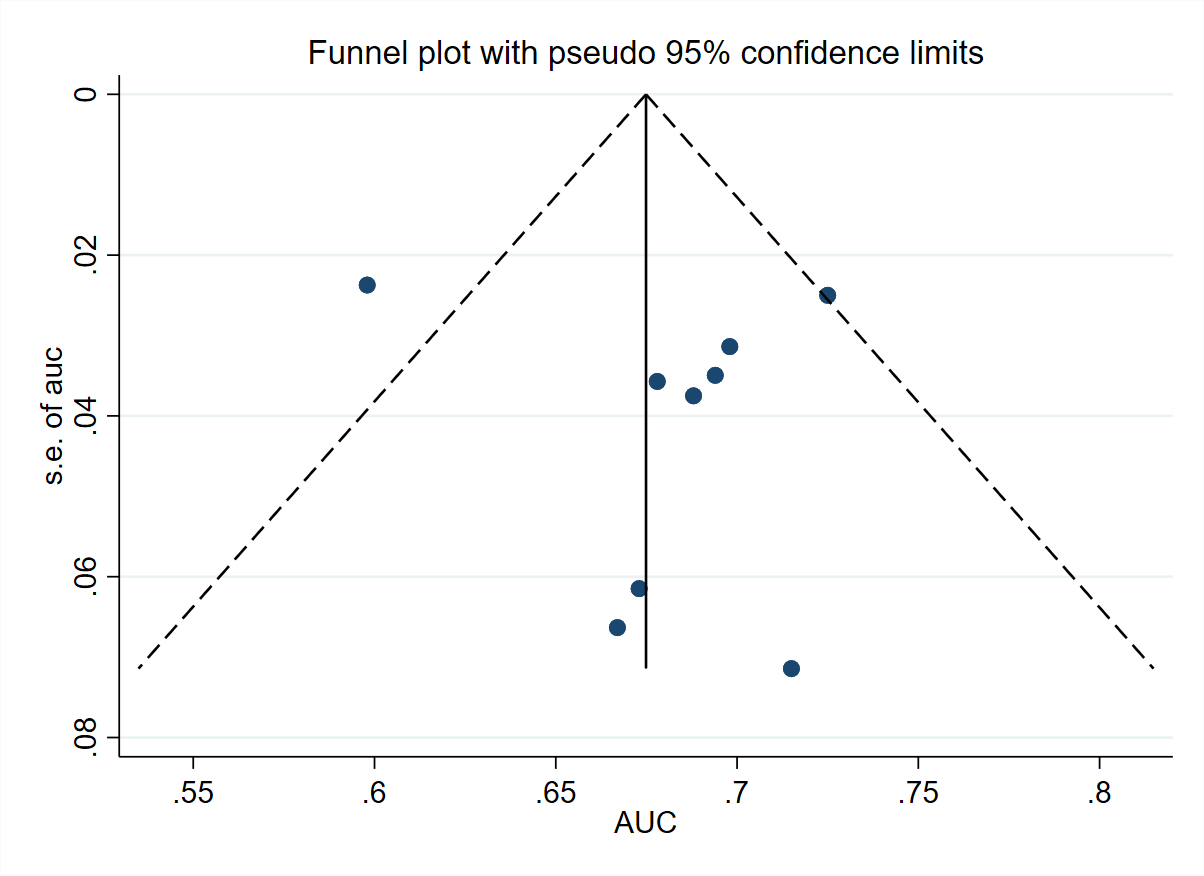

Supplement: Supplemental Material [file IANN_A_2612820_SM3028.zip › suppl_data/Supplementary Figure 3.tiff]

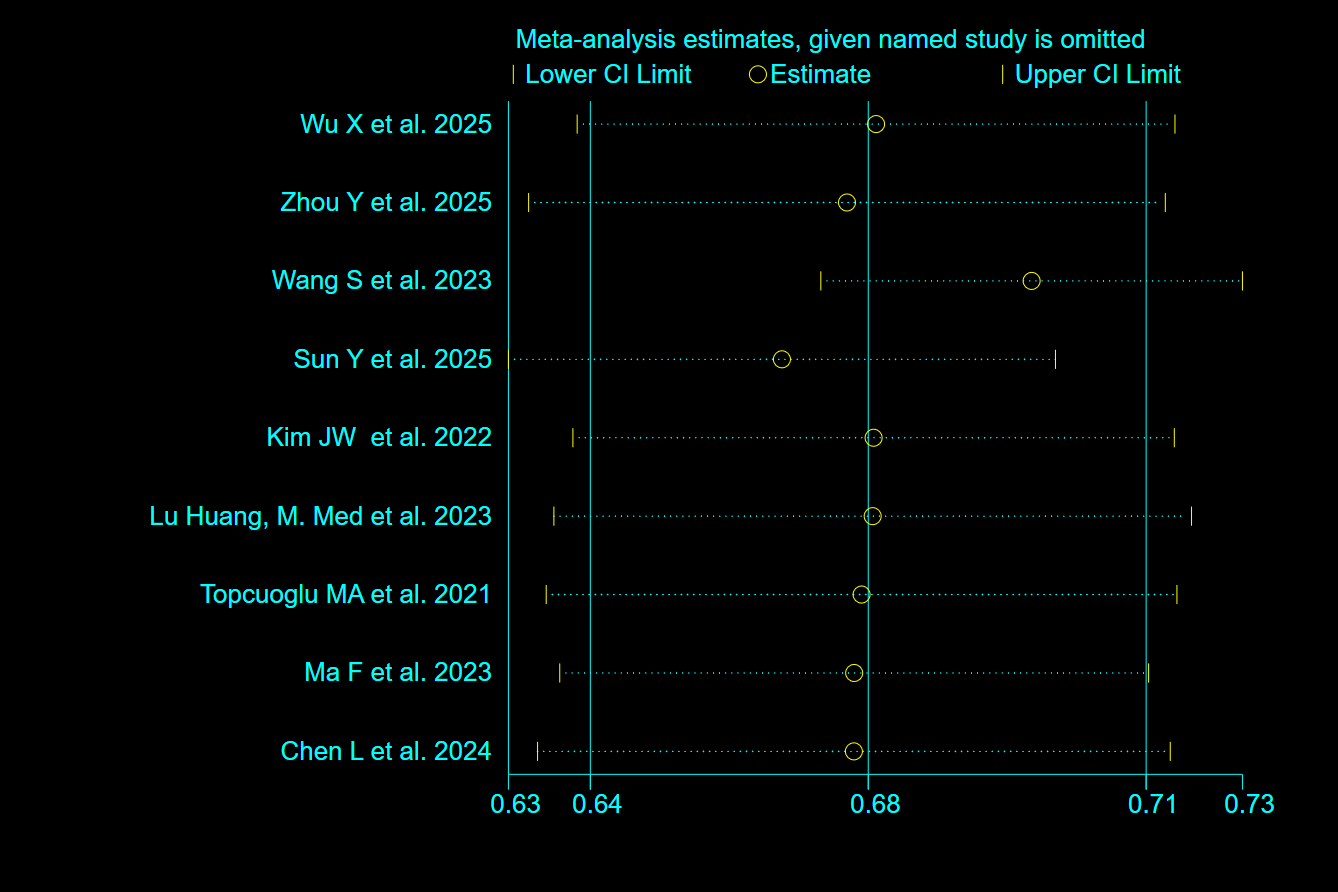

Supplement: Supplemental Material [file IANN_A_2612820_SM3028.zip › suppl_data/Supplementary Figure 4.tiff]

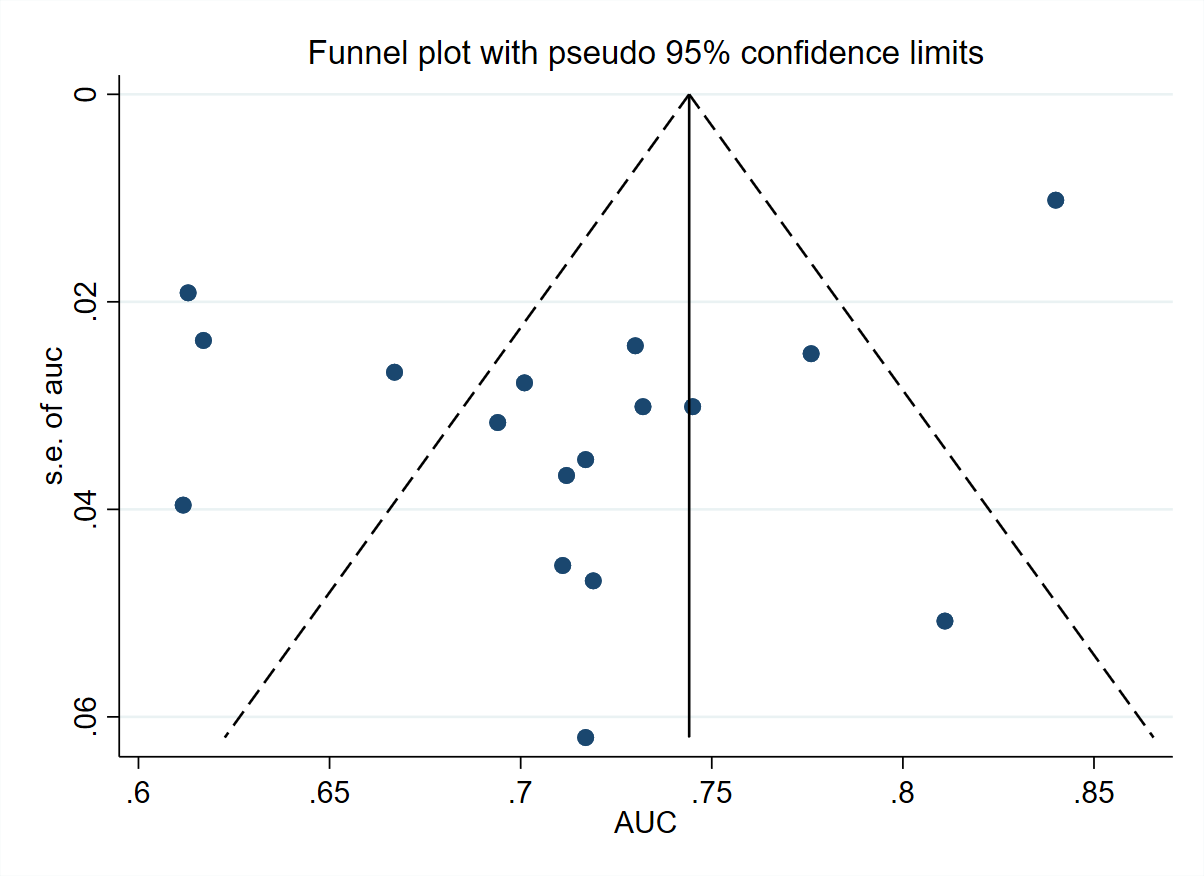

Supplement: Supplemental Material [file IANN_A_2612820_SM3028.zip › suppl_data/Supplementary Figure 5.tiff]

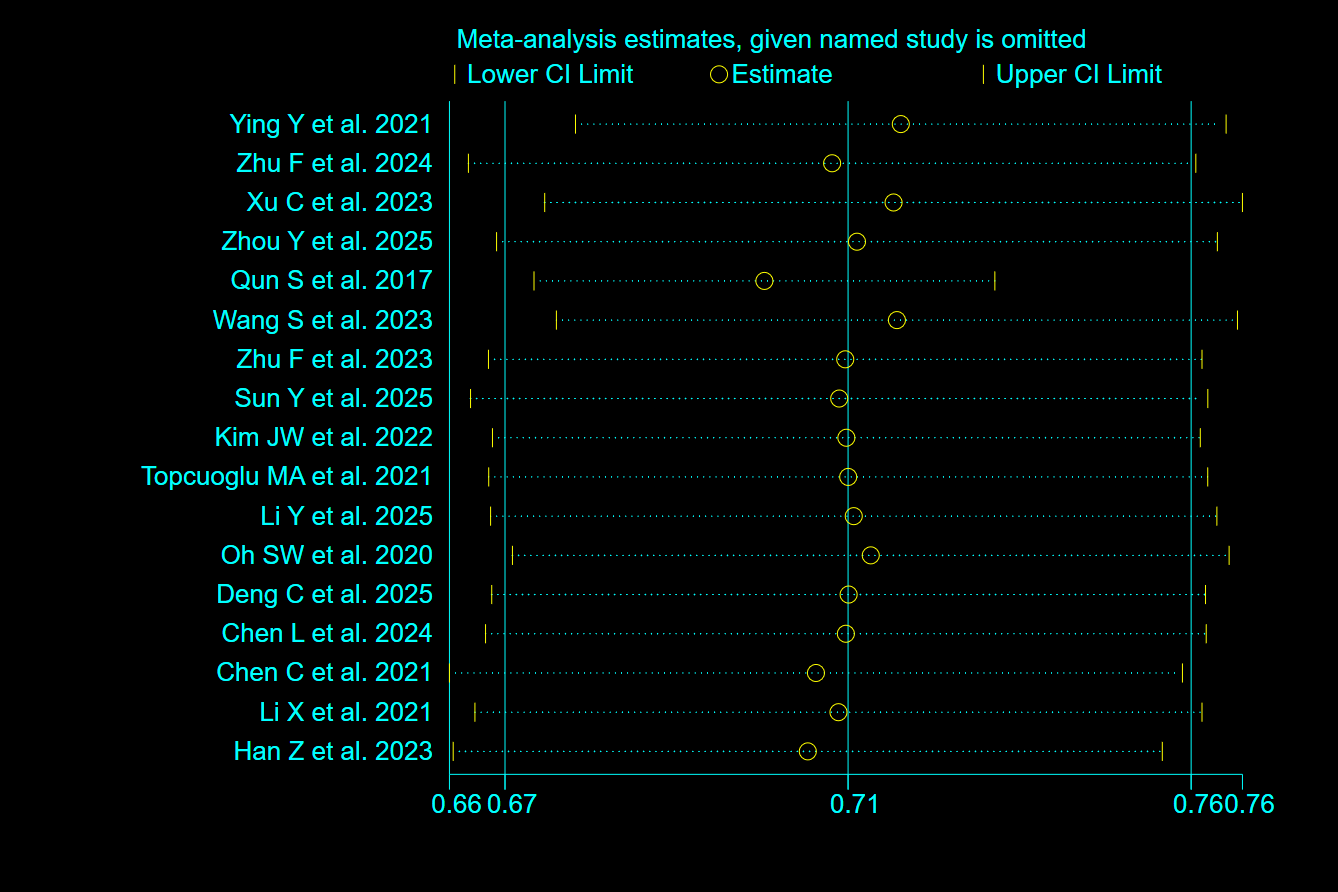

Supplement: Supplemental Material [file IANN_A_2612820_SM3028.zip › suppl_data/Supplementary Figure 6.tiff]

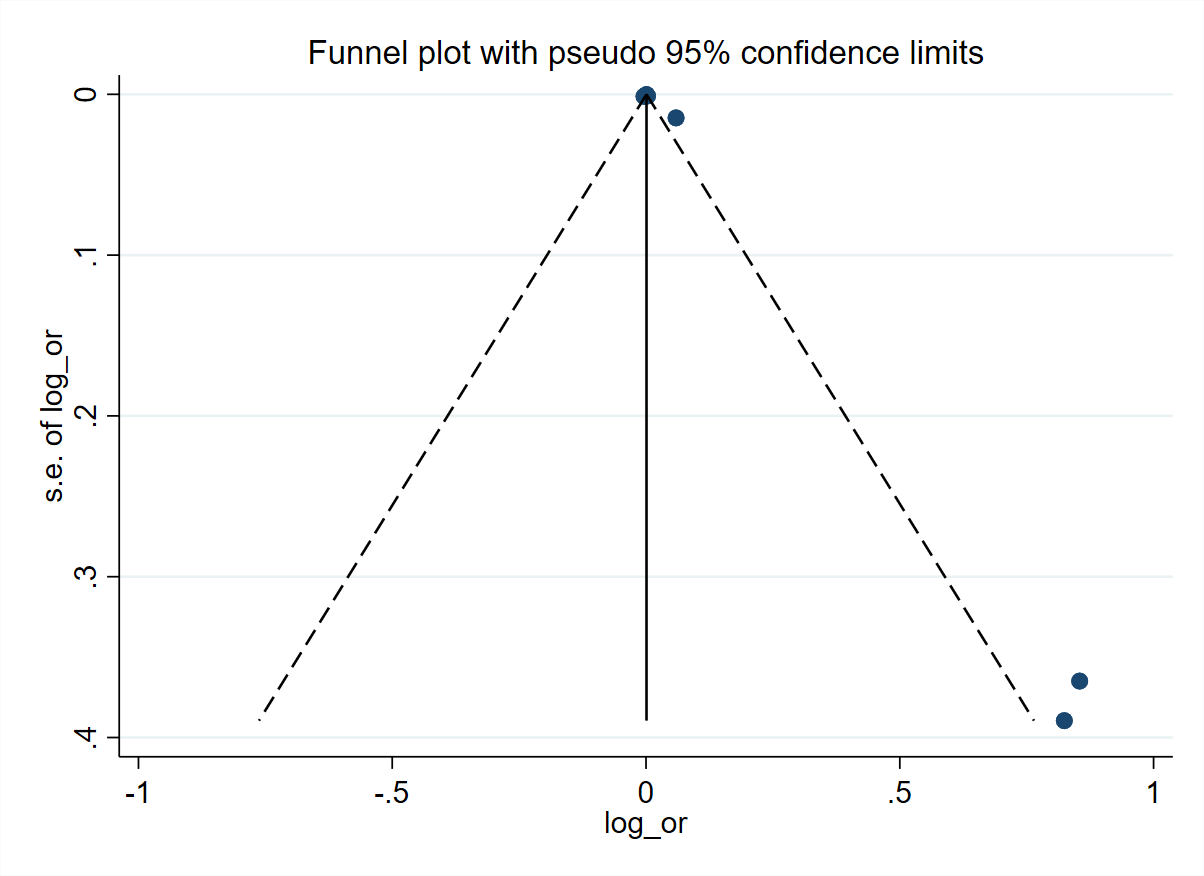

Supplement: Supplemental Material [file IANN_A_2612820_SM3028.zip › suppl_data/Supplementary Figure 7.tiff]

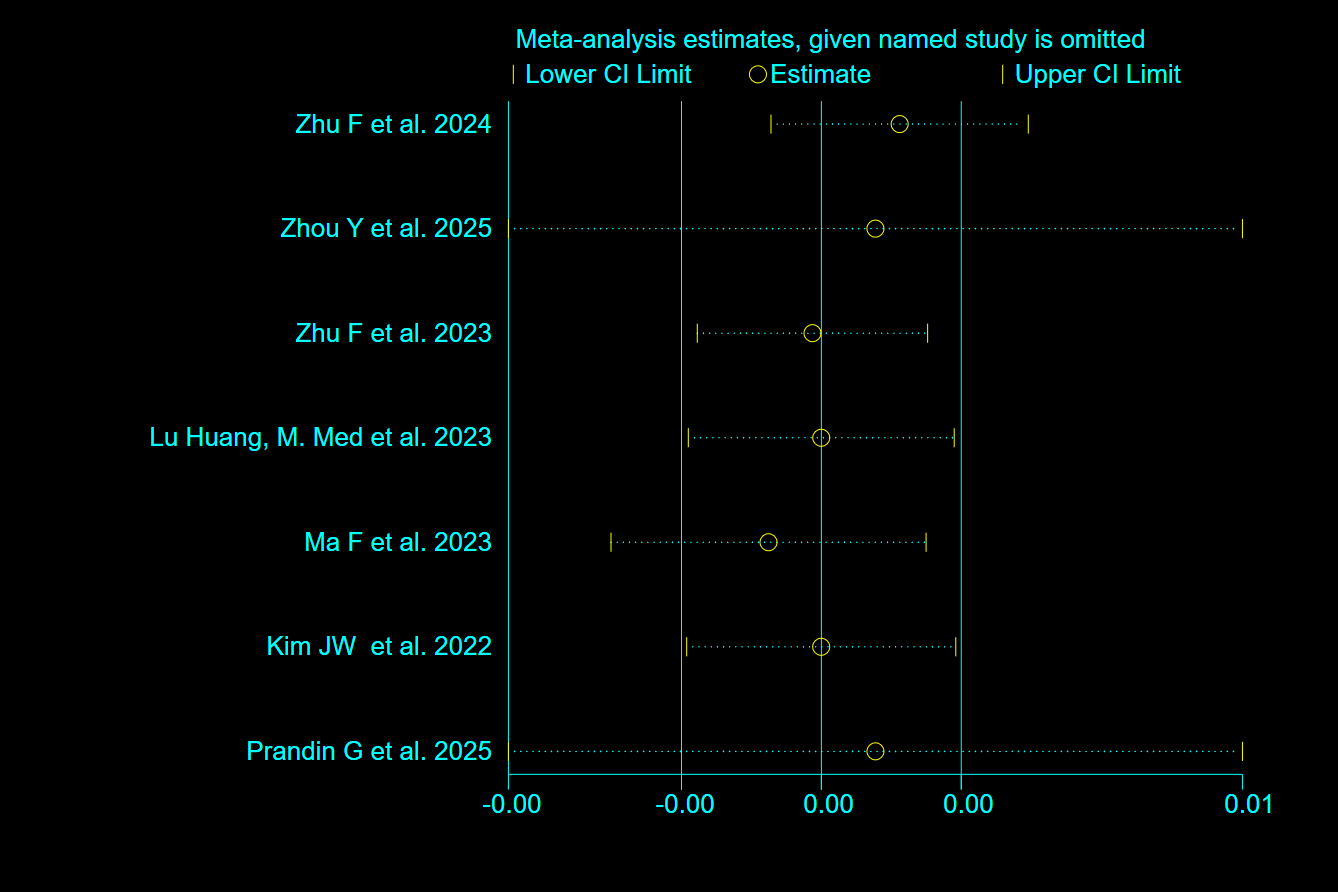

Supplement: Supplemental Material [file IANN_A_2612820_SM3028.zip › suppl_data/Supplementary Figure 8.tiff]

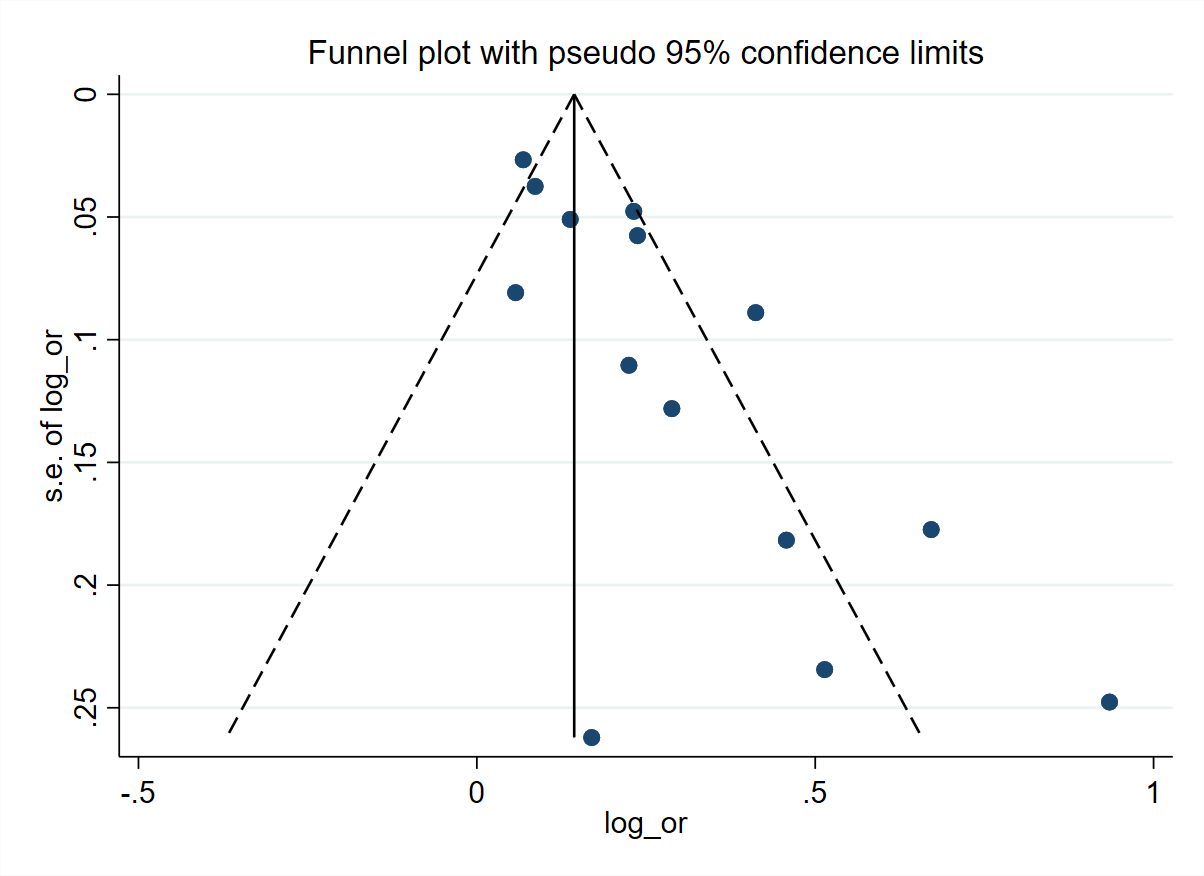

Supplement: Supplemental Material [file IANN_A_2612820_SM3028.zip › suppl_data/Supplementary Figure 9.tiff]
